# Onset of microglial entry into developing quail retina coincides with increased expression of active caspase-3 and is mediated by extracellular ATP and UDP

**DOI:** 10.1371/journal.pone.0182450

**Published:** 2017-08-01

**Authors:** María Martín-Estebané, Julio Navascués, Ana Sierra-Martín, Sandra M. Martín-Guerrero, Miguel A. Cuadros, María-Carmen Carrasco, José L. Marín-Teva

**Affiliations:** 1 Departamento de Biología Celular, Facultad de Ciencias, Universidad de Granada, Granada, Spain; 2 Departamento de Biología Celular, Fisiología e Inmunología, Facultad de Biociencias, Universidad Autónoma de Barcelona, Bellaterra, Barcelona, Spain; Instituto Cajal-CSIC, SPAIN

## Abstract

Microglial cell precursors located in the area of the base of the pecten and the optic nerve head (BP/ONH) start to enter the retina of quail embryos at the 7^th^ day of incubation (E7), subsequently colonizing the entire retina by central-to-peripheral tangential migration, as previously shown by our group. The present study demonstrates a precise chronological coincidence of the onset of microglial cell entry into the retina with a striking increase in death of retinal cells, as revealed by their active caspase-3 expression and TUNEL staining, in regions dorsal to the BP/ONH area, suggesting that dying retinal cells would contribute to the microglial cell inflow into the retina. However, the molecular mechanisms involved in this inflow are currently unclear. Extracellular nucleotides, such as ATP and UDP, have previously been shown to favor migration of microglia towards brain injuries because they are released by apoptotic cells and stimulate both chemotaxis and chemokinesis in microglial cells via signaling through purinergic receptors. Hence, we tested here the hypothesis that ATP and UDP play a role in the entry and migration of microglial precursors into the developing retina. For this purpose, we used an experimental model system based on organotypic cultures of E6.5 quail embryo retina explants, which mimics the entry and migration of microglial precursors in the *in situ* developing retina. Inhibition of purinergic signaling by treating retina explants with either apyrase, a nucleotide-hydrolyzing enzyme, or suramin, a broad spectrum antagonist of purinergic receptors, significantly prevents the entry of microglial cells into the retina. In addition, treatment of retina explants with either exogenous ATP or UDP results in significantly increased numbers of microglial cells entering the retina. In light of these findings, we conclude that purinergic signaling by extracellular ATP and UDP is necessary for the entry and migration of microglial cells into the embryonic retina by inducing chemokinesis in these cells.

## Introduction

Microglia are resident macrophages of the central nervous system (CNS) that derive from myeloid hematopoietic progenitors [1–3]. They fulfill crucial functions in the construction of the complex architecture and circuitry of the CNS during embryonic development (reviewed in [4–6]). Over the past few years, the utilization of genetic inducible fate mapping techniques in mice has revealed that microglia originate from yolk sac-derived primitive macrophages that colonize the brain rudiment at very early stages of embryogenesis and persist in the adult brain [1, 3, 7, 8], where they self-maintain by local proliferation [9, 10]. In the zebrafish, however, embryonic microglia are of extraembryonic origin, as in the mouse, but the ventral wall of the dorsal aorta is the intraembryonic source of adult microglia [11]. A further instance of the dual origin of microglia was observed in experiments in which genetically labeled yolk-sac derived blood cells were injected into the bloodstream of chick embryos; the results supported the yolk sac origin of embryonic microglia in birds but reported their replacement during posthatch development by microglia derived from an intraembryonic source [12]. Regardless of the origin of microglia during development and in adulthood, it is beyond doubt that yolk sac-derived microglial progenitors enter the CNS at early stages of the vertebrate embryonic development and spread throughout the CNS to become microglia. Once inside the CNS, microglial progenitors are called amoeboid microglia [13], which move by tangential and radial migration to reach their final destinations within the nervous parenchyma, where they differentiate into ramified microglia (reviewed in [14]). However, the molecular mechanisms responsible for the entry of microglial progenitors into the developing CNS are poorly understood.

Our previous studies showed the developmental program of microglia in the quail embryo retina [15–18]. Thus, microglial precursors enter the retina from the region occupied by the base of the pecten and the optic nerve head (BP/ONH), starting at the 7^th^ day of incubation (E7). Then, amoeboid microglia colonize the entire retina by tangential migration in a central-to-peripheral direction. Subsequent radial migration in a vitreal-to-scleral direction allows amoeboid microglia to reach the plexiform layers, where they differentiate into ramified microglia. Other studies in our lab showed that *in vitro* cultures of quail embryo retina explants mimic the migration and differentiation of microglial precursors in the *in situ* developing retina [19, 20]. Therefore, these organotypic cultures of retina explants represent an excellent experimental model system to investigate possible molecular factors involved in the entry of microglial precursors into the retina.

Numerous studies have highlighted the temporal and spatial coincidence between microglial immigration and programmed cell death (PCD) during the normal development of the vertebrate CNS [16, 21–27], related to the capacity of microglia for apoptotic cell clearance. In fact, it has been well documented in different regions of the developing CNS that immigrant microglia are involved in the phagocytosis of dying cells, participating in the clearance of apoptotic corpses in cell death regions (for review, see [24, 28]). The developing CNS must possess mechanisms that guide microglia towards apoptotic targets in order to achieve the effective removal of cell debris. These mechanisms would include “find-me” signals released by apoptotic cells in their earliest stages of death, which would direct the migration of distant microglia. The chemokine CX3CL1 (fractalkine), the lipid lysophosphatidylcholine, and the nucleotides ATP/ADP and UTP/UDP have been reported to be the main molecules acting as “find-me” signals released by dying cells to attract macrophages (for review, see [29, 30]), and they may also guide microglia toward apoptotic neurons [4, 24, 28]. In this way, PCD in nervous regions devoid of microglia in early developmental stages may be responsible for releasing “find-me” signals that guide the entry of microglial precursors into the CNS and thereby initiate the microglial colonization process. PCD would also strengthen microglial colonization of the CNS by triggering microglial proliferation, as shown in the developing mouse forebrain [31].

Abundant research has shown that ATP/ADP and UTP/UDP are “find-me” signals that can stimulate both chemotaxis and chemokinesis in microglial cells, attracting them to sites of neuronal damage in the injured adult brain [32–39]. Microglia can sense ATP/ADP and UTP/UDP leaked from injured cells through P2X and P2Y purinergic receptors, and they react by extending processes and migrating toward the injured sites [40–43]. Until recently, however, it was not clear whether this microglial recruitment mechanism also functions during normal CNS development. Two recent studies in the developing zebrafish revealed that microglial colonization of the brain is triggered by developmental neuronal apoptosis [44, 45]. The two studies differed in their focus on the specific molecular factor involved in the microglial recruitment, finding that nucleotide [44] and lysophosphatidylcholine [45] signaling both appear to play a role in attracting microglial progenitors into the developing zebrafish brain. However, no data are yet available on the molecular factors that trigger entry of microglial progenitors into the CNS during the development of avian embryos.

We demonstrate here a chronological coincidence between the onset of microglial cell entry into the quail embryo retina and a striking increase in the death of retinal cells in regions dorsal to the BP/ONH area. We also used our *in vitro* model system based on organotypic cultures of quail embryo retina explants to show that purinergic signaling by extracellular ATP and UDP, presumably released from dying retinal cells, is involved in the entry and chemokinetic migration of microglial cells into the retina, similar to observations in the developing zebrafish brain. Hence, this mechanism for microglial colonization of the CNS appears to be a conserved process across vertebrate species.

## Materials and methods

### Animals

Eyes from quail (*Coturnix coturnix japonica*) embryos at E6 and E7 were used to study the relationship between the first entry of microglial cells into the *in situ* retina and the apoptotic cell death taking place during these stages of retinal development. In addition, organotypic cultures of explants from E6.5 quail retinas were used to examine molecular mechanisms that control the first entry of microglial cells into the retina.

Experimental procedures were approved by the Animal Experimentation Ethics Committee of the University of Granada and followed the guidelines of the European Union Directive 2010/63/EU on the protection of animals used for scientific purposes.

### Organotypic cultures of E6.5 retina explants

Explants from E6.5 retinas were cultured *in vitro* on Millicell culture plate inserts according to the method described by Stoppini et al. [46] with some modifications [19]. Retinas were dissected out into cold Gey’s balanced salt solution (Sigma, St. Louis, MO) supplemented with 5 mg/mL glucose (Sigma) and 50 IU-μg/mL penicillin-streptomycin (Invitrogen, Paisley, United Kingdom). After removing the pigment epithelium, square explants (3 mm x 3 mm) were isolated from the central area of each retina and then placed on Millicell inserts (Millicell CM, 30 mm, pore size 0.4 μm, Millipore, Bedford, MA) vitreal surface down. Millicell inserts were placed in six-well plates containing 1 mL/well culture medium composed of 50% basal medium with Earle’s salts, 25% Hank’s balanced salt solution, 25% horse serum, 1 mM L-glutamine, 10 IU-μg/mL penicillin-streptomycin (all purchased from Invitrogen), and 5 mg/mL glucose. E6.5 retina explants were then incubated at 37°C in a humidified atmosphere with 5% CO_2_ for 24 hours *in vitro* (E6.5+24hiv retina explants).

### Retina explant treatments

E6.5 retina explants were subjected to experimental treatments with different reagents (as described below), which were added to the culture medium from the beginning of *in vitro* incubation. In each experiment, reagent treatment was carried out on the retina explant obtained from the right eye of each quail embryo, whereas the retina explant from the left eye of the same embryo was cultured in reagent-free medium and served as a control. Apyrase (Sigma, no. A6132), a potato enzyme that catalyzes the hydrolysis of extracellular tri- and di-phosphate nucleotides, was used at 10U/ml. Suramin sodium salt (Suramin, Sigma, no. S2671), a broad spectrum antagonist of the P2X and P2Y purinergic receptors, was used at 100 μM. Adenosine 5′-triphosphate disodium salt (ATP, Sigma, no. A6419) was added to the culture medium at 10 μM, 100 μM, and 1mM. Adenosine 5′-(3-thiotriphosphate) tetralithium salt (ATPγS, Sigma, no. A1388), a non-hydrolyzable ATP analogue, was used at 100 μM. Uridine 5´-diphosphate disodium salt hydrate (UDP, Sigma, no. 94330) was added at 100 μM and 1mM. Q-VD-OPh (R&D Systems, Minneapolis, MN, no. OPH001), a broad spectrum caspase inhibitor, was used at 100 μM.

### Cell viability assay

Cell viability in reagent-treated and control E6.5+24hiv retina explants was determined by flow cytometry of cell suspensions obtained by dissociation of retinal explants, using fluorescein diacetate (FDA) and propidium iodide (PI).

After their culture, retinal explants were rinsed in 0.01M phosphate buffered saline (PBS) and dissociated in 1 ml PBS using a Dounce homogenizer (Pobel, Madrid, Spain). Then, the resulting suspensions were incubated for 15 minutes with 10 μg/ml PI (Sigma) and 15 μg/ml FDA (Sigma) in the dark. Samples were analyzed by flow cytometry using FACS Canto II flow cytometer (Beckton Dickinson, Franklin Lakes, NJ). Cell viability was determined in each sample as the percentage of FDA-positive cells with respect to the total cell number (FDA-positive plus IP-positive cells). The mean cell viability was determined in at least six reagent-treated E6.5+24hiv retina explants and their corresponding controls for each treatment.

### Immunocytochemistry

Microglial cells were identified in cultured retina explants and non-cultured retinas by immunolabeling with the monoclonal antibody QH1 (Developmental Studies Hybridoma Bank [DSHB], University of Iowa, Iowa City, IA), which recognizes all quail hemangioblastic cells except for mature erythrocytes [47], including amoeboid, ramified, and activated microglia [48]. Active caspase-3 was identified by immunolabeling with an anti-active caspase-3 polyclonal antibody (R&D System). The monoclonal antibodies H5 and 39.4D5 (DSHB) were used to recognize the vimentin cytoskeleton of Müller cells and cells expressing transcription factor Islet-1, respectively.

Double QH1/anti-active caspase-3 immunolabeling was carried out on whole-mounted non-cultured retinas, which were fixed in paraformaldehyde-lysine-periodate (PLP) [49] for 1 hour and permeabilized in PBS containing 0.1% Triton X-100 (PBS-Tr) for 4 hours. They were subsequently incubated overnight at 4°C in a mix of polyclonal anti-active caspase-3 and monoclonal antibody QH1. Both primary antibodies were diluted (1:100 and 1:4, respectively) in 1% bovine serum albumin in 0.01M PBS (BSA-PBS) containing 0.25% Triton X-100 (BSA-PBS-Tr). After rinsing in PBS-Tr, whole-mounted retinas were incubated for 4 hours at room temperature in the secondary antibodies Alexa Fluor 594-conjugated goat anti-rabbit IgG and Alexa Fluor 488-conjugated goat anti-mouse IgG (Molecular Probes, Eugene, OR) diluted 1:500 in BSA-PBS-Tr, rinsed again, and coverslipped with Fluoromount G (Southern Biotech, Birmingham, AL) with the vitreal side up.

Cross cryosections of non-cultured retinas were also used for double QH1/anti-active caspase-3 immunolabeling. Specimens were fixed for 1 hour at 4°C in PLP, thoroughly rinsed in PBS-Tr, cryoprotected overnight at 4°C in 20% sucrose in PBS-Tr, and embedded into 7.5% gelatin and 20% sucrose in PBS-Tr. Solidified blocks containing the specimens were then embedded in OCT compound, frozen in isopentane cooled with liquid nitrogen, and stored at -40°C before sectioning on a Leica CM1850 cryostat. Twenty micrometer-thick cryosections were obtained on Superfrost slides (Menzel-Glasser, Braunschweig, Germany), hydrated in PBS, and treated for double QH1/anti-active caspase-3 immunolabeling following a similar schedule to that described for whole-mounts, with minor modifications (cryosections were not permeabilized, and the incubation in secondary antibodies was reduced to 2.5 hours). In addition, cell nuclei were stained in immunolabeled cryosections with the nuclear dye Hoechst 33342 (Sigma).

Whole-mounted E6.5+24hiv retina explants underwent single QH1 immunolabeling or double QH1/anti-active caspase-3 antibody immunolabeling, following a similar protocol to that described for whole-mounted non-cultured retinas. Cross cryosections of control and reagent-treated E6.5+24hiv retina explants were also used for single anti-active caspase-3 (1:100), H5 (1:40) and 39.4D5 (1:100) immunolabeling to test whether experimental treatments did or did not produce structural alterations in the explants.

### TUNEL staining

The localization of apoptotic cells observed in E6 and E7 non-cultured retinas by anti-active caspase-3 immunofluorescence was compared with that demonstrated by TUNEL staining. Hydrated and permeabilized cross cryosections were treated for 1 hour at 37°C with a solution containing 0.01 units/μl of terminal deoxynucleotidyl transferase enzyme (TdT, Promega, Madison, WI) in TdT buffer (Promega) and 4 nmol/ml biotin-16-dUTP (Roche Diagnostics, Mannheim, Germany). After incubation, sections were washed with PBS and incubated in Alexa Fluor 488-conjugated streptavidin (Molecular Probes) for 1 hour at room temperature. Following a blocking step in 10% NGS-PBS-Tr, sections underwent single anti-active caspase-3 (1:100) immunolabeling, using the protocol described above. Cell nuclei were stained with the nuclear dye Hoechst 33342.

The proportion of active caspase-3-positive cells colabeled with TUNEL was determined by counts (with 40X objective) in specific microscopic fields in the dorsal part of double anti-active caspase-3/TUNEL-labeled histological sections from E6 and E7 non-cultured retinas (three retinas per age). These microscopic fields spanned 300 μm in width and the entire retinal thickness in height and were selected from 10 histological sections per retina, collected at intervals of 200 μm along the anterior-posterior axis of the eye. Results were expressed as the percentage of caspase-3-positive cells colabeled with TUNEL with respect to the total number of caspase-3-positive cells.

### Quantification of active caspase-3 positive cells by flow cytometry

Active caspase-3 positive cells were quantified by flow cytometry of cell suspensions obtained from reagent-treated and control E6.5+24hiv retina explants labeled with anti-active caspase-3 antibody. Explants were fixed in 4% paraformaldehyde for 15 minutes, rinsed in PBS, and incubated overnight at 4°C in anti-active caspase-3 antibody (1:100). After rinsing in PBS, retina explants were incubated for 1 hour at room temperature in the secondary antibody Alexa Fluor 488-conjugated goat anti-rabbit IgG and subsequently dissociated in 1 ml PBS. After obtaining a cell suspension, the percentage of caspase-3 positive cells was determined by flow cytometry with respect to the total cell number (10,000 events) analyzed in at least six reagent-treated E6.5+24hiv retina explants and their corresponding controls for each treatment.

In order to test the correlation between cell death and the entry of microglial cells into the retina, caspase-3-positive cells and microglial cells in the retina were simultaneously quantified in E6.5+24hiv retina explants treated with the caspase inhibitor Q-VD-OPh and in their respective controls. In brief, after fixation, each explant was incubated for 1 hour at room temperature in Alexa Fluor 594-conjugated QH1 antibody (1:500) and extended on a slide to obtain micrographs for subsequent microglial cell quantification. Then, the explant was rinsed in PBS, and the above protocol was followed for the quantification of caspase-3-positive cells by flow cytometry.

### Determination of microglial proliferation by BrdU labeling

Microglial proliferation in ATP- and UDP-treated E6.5+24hiv retina explants and their respective controls was detected by adding 5-bromo-2’-deoxyuridine (BrdU) (Sigma) to the medium at a final concentration of 10 μM for the last 12 hours of *in vitro* culture, given that microglial cells enter the retina from E7 onward. Retina explants were then fixed in PLP for 1 hour and rinsed in PBS, followed by DNA denaturation with 2N HCl for 1 hour at 37°C and neutralization with 0.1 M Na borate (pH 8.5) for 25 minutes at room temperature. They were blocked for 15 minutes in PBS-Tr containing 5% normal goat serum (NGS) at room temperature and incubated overnight at 4°C in a mix of monoclonal anti-BrdU (1:800) (Sigma) and polyclonal anti-iNOS (1:500) (Abcam, Cambridge, United Kingdom) antibodies, both diluted in BSA-PBS-Tr. Polyclonal anti-iNOS antibody was used as microglial marker, because amoeboid microglia express iNOS during the normal embryonic development of quail retina [20]. The explants were subsequently rinsed in PBS-Tr and incubated with the secondary antibodies Alexa Fluor 594-conjugated goat anti-mouse IgG and Alexa Fluor 488-conjugated goat anti-rabbit IgGs for 2 hours at room temperature. Finally, retina explants were coverslipped with Fluoromount G with the vitreal side up.

The percentage of proliferating cells was evaluated by counting BrdU-positive microglial cell nuclei and calculating their percentage with respect to the total number of microglial cells in at least eight reagent-treated explants and their corresponding controls for each treatment.

### Microscopy

Observations of fluorescent specimens were made with a Leica TCS-SP5 confocal microscope (Leica, Wetzlar, Germany). Stacks of confocal optical sections of selected microscopic fields were collected at 0.5–1 μm intervals and projection images were obtained, stored in TIFF format, and digitally prepared with Adobe Photoshop (Adobe Systems, San José, CA).

### Quantitative analysis of numbers and morphological features of microglial cells in E6.5+24hiv retina explants

QH1-labeled microglial cells that had entered the retina were counted on microscopic fields of 2 mm x 2 mm in each whole-mounted E6.5+24hiv retina explant. Mean values of microglial cell numbers were determined from counts made in at least 10 reagent-treated explants and their corresponding controls.

Indicators of morphological features of QH1-labeled microglial cells were determined in reagent-treated and control E6.5+24hiv retina explants by morphometric analysis using Image Tool 2.0 software (University of Texas Health Science Center, San Antonio, TX). Cell profile area and cell elongation index were used as indicators to assess changes in the migratory phenotype of microglial cells after treatment with different reagents. The elongation index of a cell was defined as the ratio of its major axis length to its minor axis length. The mean values of these cell parameters were determined in at least 10 reagent-treated E6.5+24hiv retina explants and their corresponding controls, based on data obtained in three different square microscopic fields of 0.25 mm^2^ (500 μm x 500 μm) in each specimen.

### Statistical analysis

Data are reported as means ± standard error of the mean (SEM). Statistical differences between reagent-treated retina explants and their controls were determined with the Student´s t-test. All analyses were performed using IBM SPSS statistics software version 20.0.0 for Windows (Chicago, IL, USA). Differences were considered significant at P<0.05.

## Results

### Initiation of microglial cell colonization of the quail retina at E7 coincides with a marked increase in active caspase-3 expression

**Fig 1:**
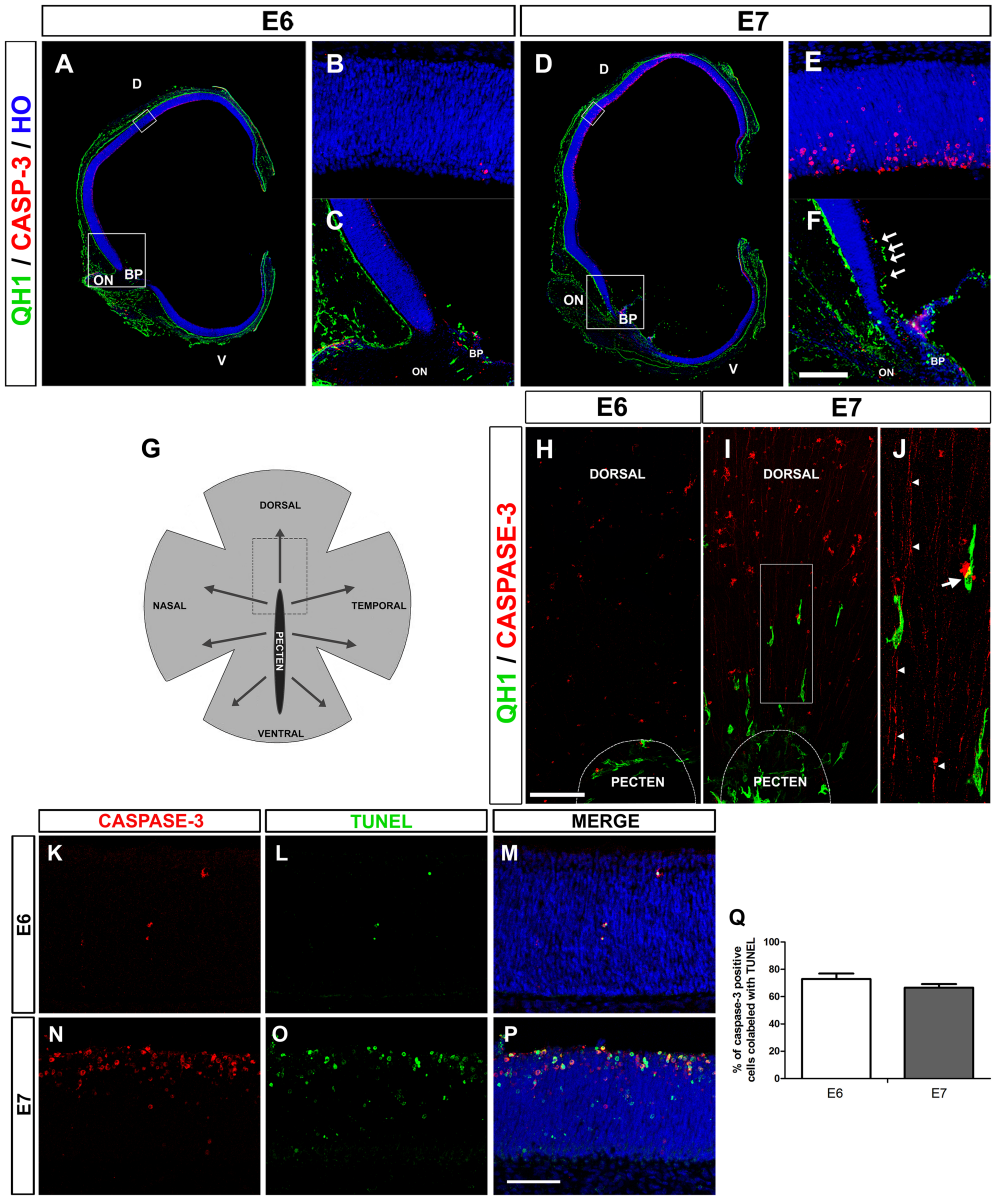
The onset of microglial entry into the embryonic quail retina coincides with an increase in the death of retinal cells. **(A, D)** Confocal images of QH1 (green) and anti-active caspase-3 (red) double-immunostained cross sections from quail embryo eyes at 6 (E6, **A**) and 7 (E7, **D**) days of incubation. Cell nuclei are stained with Hoechst 33342 (blue). D: dorsal; V: ventral; BP: base of the pecten; ON: optic nerve. (**B, E)** Higher magnifications of the upper boxed areas in **A** and **D**, respectively. Active caspase-3-expressing cells (dying cells) are almost non-existent in the E6 retina (**B**) and are markedly increased in the dorsal region of the E7 retina (**E**). (**C, F)** Higher magnifications of the lower boxed areas in **A** and **D**, respectively. No QH1-positive microglial cells are seen in the retina at E6 (**C**), whereas some (arrows) are migrating within the retina at E7 in the vitreal part of the region dorsal to the ON and BP (**F**). Scale bar in **F**: 725 μm for **A** and **D**; 50 μm for **B** and **E**; 160 μm for **C** and **F**. (**G**) Schematic drawing representing a whole-mounted quail embryo retina, with the base of the pecten (black area) in a central location, from which microglia migrate in a central-to-peripheral direction (arrows) from 7 days of incubation (E7) onwards. The area delimited with dashed lines represents the retinal zone immediately dorsal to the pecten, shown in **H** and **I**. (**H-J**) Confocal images of the region dorsal to the base of the pecten (BP) of QH1 (green) and anti-active caspase-3 (red) double-immunostained whole-mounted quail embryo retinas at E6 (**H**) and E7 (**I**). No microglial cells are present within the E6 retina, in which caspase-3-positive dying cells are almost absent (**H**). By contrast, at E7, some elongated microglial cells enter the retina from the BP and are directed toward a retinal area with numerous caspase-3-positive cells (**I**). Higher magnification of the boxed area in **I** is displayed in **J**, showing the close contact between a migrating microglial cell and the soma of a caspase-3-positive cell (arrow). Another microglial cell is in close contact with long thin caspase-3-positive processes (arrowheads) that appear to be axons from dying cells. Scale bar in **H**: 100 μm for **H** and **I**; 37 μm for **J**. (**K-P**) Anti-active caspase-3 (red) and TUNEL (green) double-labeled cross-sections from E6 (**K-M**) and E7 (**N-P**) retinas. The co-localization of anti-active caspase-3 immunolabeling and TUNEL staining in numerous cells demonstrates that they are apoptotic cells. Scale bar in **P**: 50 μm for **K-P**. (**Q)** Percentage of active caspase-3 positive cells colabeled with TUNEL in E6 (white bar) and E7 (grey bar) retinas. Data are expressed as means ± SEM (n = 3 for each age). Despite the much higher presence of active caspase-3-positive cells in E7 than in E6, the percentage of caspase-3/TUNEL-positive cells is very similar in each developmental stage.

Previous studies of our group showed that amoeboid microglial cells enter the retina of quail embryos from the BP/ONH area between E7 and hatching (E16) and then migrate tangentially in a central-to-peripheral direction on the vitreal part of the embryonic retina [15–17]. In the present study, immunofluorescence analysis revealed that active caspase-3 expression was very low in the quail embryo retina at E6 (Fig 1A and 1B), when microglial cells had not begun to enter the retina (Fig 1C), but was markedly increased at E7 (Fig 1D and 1E), when the first microglial cells were entering the retina from the BP/ONH area (Fig 1F). Active caspase-3-positive cells were located mainly in the inner half of the neuroblastic layer of the retina, where the optic nerve fiber layer, ganglion cell layer (GCL) and inner plexiform layer would form. The chronotopographical coincidence of the beginning of active caspase-3 expression with the onset of microglial cell entry into the retina was clearly observed at E7 in the central region of the retina, located dorsal to the BP/ONH area (Fig 1G–1J). At E6, very few active caspase-3-positive cells were seen in this region when microglial precursors were restricted to the BP/ONH area (Fig 1H). At E7, however, an evident increase in the number of active caspase-3-positive cells was observed dorsal to the BP/ONH, coinciding with the presence of recently-arrived microglial cells (Fig 1I). These active caspase-3-positive cells appeared to be mainly ganglion cells, because they were localized in the vitreal part of the retina and frequently showed long thin processes directed toward the ONH, which appeared to be ganglion cell axons (Fig 1I and 1J). It is noteworthy that migrating microglial cells were frequently observed in close contact with axons and somas of active caspase-3-positive ganglion cells (Fig 1I and 1J). In addition, a high coincidence was observed between the % expression of active caspase-3 and TUNEL labeling in E6 and E7 retinas (around 70% at each age) (Fig 1K–1Q), indicating its relationship with apoptotic processes.

### Organotypic cultures of E6.5+24hiv retina explants are a reliable experimental model to study molecules regulating the early entry of microglial cells into the retina

**Fig 2:**
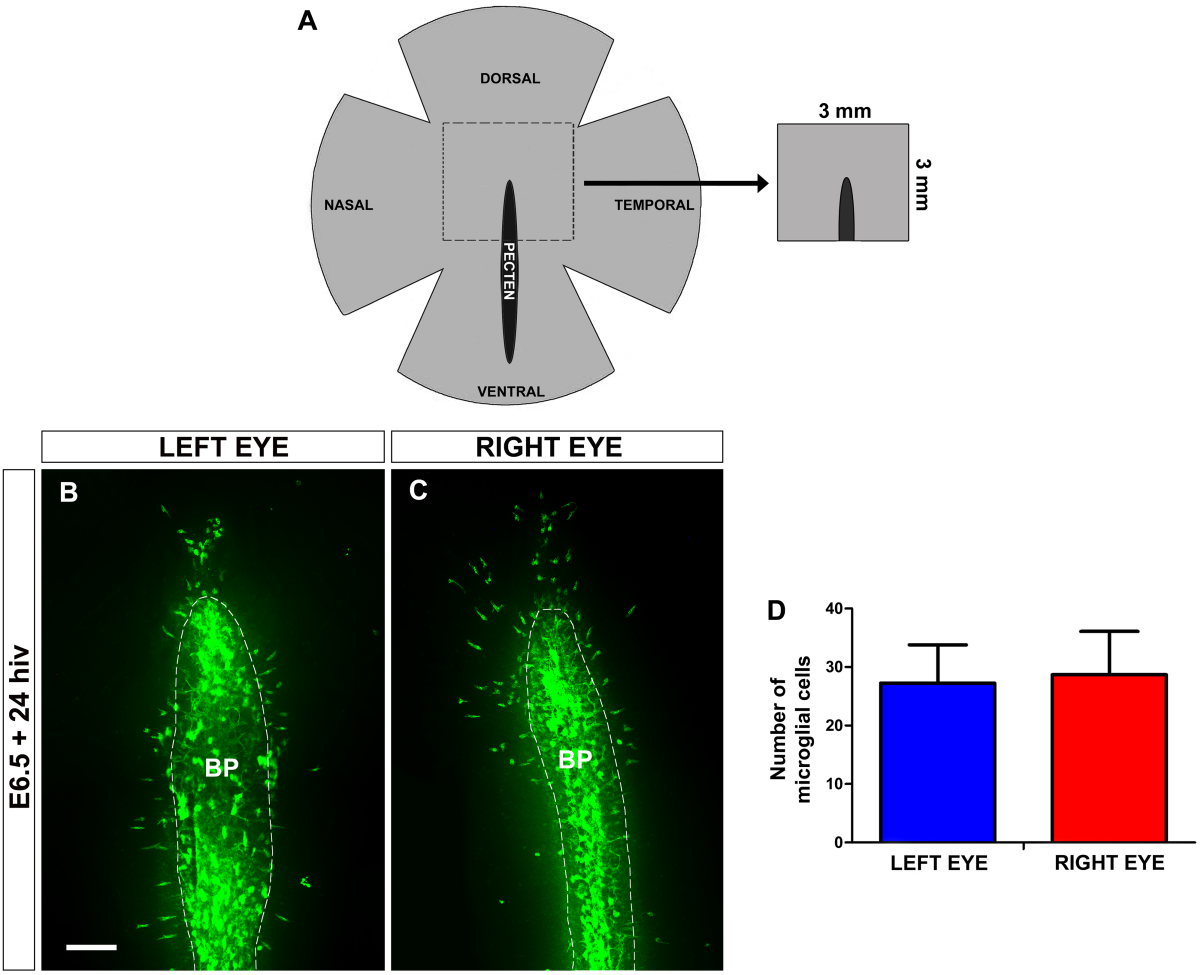
Microglial cells enter the retina at a similar rate in explants from both eyes of each quail embryo. **(A)** Schematic drawing of a whole-mounted quail embryo retina at 6.5 days of incubation (E6.5) in which the central area, delimited with dashed lines, represents a 3 mm x 3 mm retina explant including the dorsal part of the base of the pecten (black area), similar to those isolated for *in vitro* culture in this study. **(B, C)** QH1-immunolabeled (green) whole-mounted retina explants from left (**B**) and right (**C**) eyes of an E6.5 quail embryo cultured for 24 hours *in vitro* (E6.5+24hiv). Note that the number of microglial cells inside the retina is similar in the two explants. BP: base of the pecten (delimited with a dashed line). Scale bar: 100 μm. (**D**) Number of microglial cells within the retina in E6.5+24hiv quail embryo retina explants from left (blue bar) and right (red bar) eyes. Data are expressed as means ± SEM (n = 12). No significant differences are observed between left and right eyes (P>0.05, Student´s t-test).

Our group previously demonstrated that organotypic cultures of quail embryo retina explants are an excellent *in vitro* system to reproduce the physiological-like behavior of microglial cells, which follow a similar chronotopographical pattern of migration and ramification to that observed in the *in situ* developing retina [19]. In the present study, we used similar retina explants containing the dorsal part of the BP/ONH area, which were obtained from E6.5 quail embryos and cultured for 24 hiv (Fig 2A–2C), in order to test the effects of different molecules on the entry of microglial cells into the retina. The time window of development chosen for this *in vitro* study covered a period from 12 hours before to 12 hours after the first entry of microglial cells into the retina (at E7). In each quail embryo, the retina explant obtained from the right eye was used for experimental treatment, whereas the retina explant from the left eye served as non-treated control. This procedure was selected because the elongated phenotype of tangentially migrating microglial cells and their rate of entry from the BP/ONH area were similar between non-treated retina explants obtained from the two eyes in each quail embryo (Fig 2B–2D). In fact, no significant differences in the number of microglial cells entering the retina were observed between explants from the right and left eyes (Fig 2D). Therefore, E6.5+24hiv retina explants were used as a reliable *in vitro* experimental model to study factors regulating the earliest entry of microglial cells into the retina.

### Caspase inhibition in E6.5+24hiv retina explants reduces the number of active caspase-3-positive cells in parallel with a decrease in the entry of microglial cells into the retina

**Fig 3:**
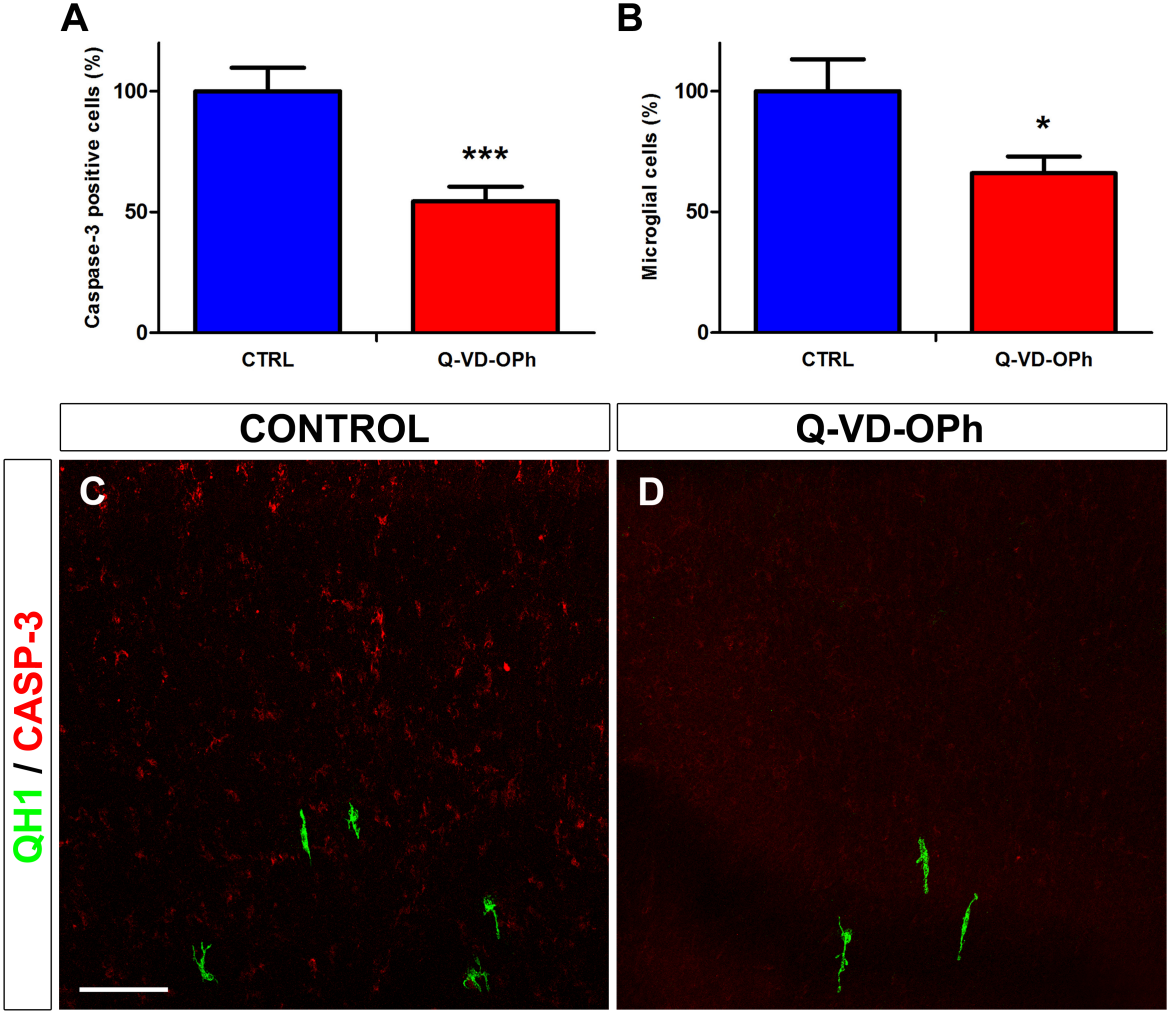
*In vitro* treatment with the broad spectrum caspase inhibitor Q-VD-OPh reduces the number of active caspase-3-positive cells in parallel with a decrease in the entry of microglial cells into the embryonic quail retina. (**A, B**) Effects of Q-VD-OPh on the numbers of active caspase-3 positive cells (**A**) and microglial cells (**B**) in retina explants from quail embryos at 6.5 days of incubation cultured for 24 hours *in vitro* (E6.5+24hiv). Quantifications of active caspase-3-positive cells (estimated by flow cytometry of cell suspensions obtained from retina explants) and QH1-positive microglial cells (counted on micrographs from the same explants as used for flow cytometry) are expressed as percentages of the respective absolute numbers in control explants (CTRL, blue bars). Percentages of caspase-3-positive cells and microglial cells are significantly lower in Q-VD-OPh-treated (Q-VD-OPh, red bars) than in non-treated (CTRL, blue bars) E6.5+24hiv retina explants, showing a relationship between cell death and the entry of microglial cells into the retina. Data are expressed as means ± SEM (n = 12 in A, n = 10 in B). Asterisks depict significant differences (*P<0.05, ***P<0.001, Student´s t-test). (**C, D**) Confocal images of the dorsal region of anti-active caspase-3 (red) and QH1 (green) double-immunostained whole-mounted E6.5+24hiv retina explants incubated in Q-VD-OPh-free (CONTROL, **C**) and 100 μM Q-VD-OPh-containing (Q-VD-OPh, **D**) medium. The presence of active caspase-3-positive cells is markedly reduced in Q-VD-OPh-treated explants. Scale bar in **C**: 50 μm for **C** and **D**.

In order to study further the relationship between the expression of active caspase-3 and the entry of microglial cells into the quail retina, E6.5 retina explants were treated during 24 hiv with the broad spectrum caspase inhibitor Q-VD-OPh (Fig 3). Although this inhibitor has been shown to be more effective than other broad spectrum caspase inhibitors, such as Boc-D-fmk or Z-VAD-fmk [50, 51], its use in our experimental system had certain limitations. In fact, the treatment of E6.5 retina explants with Q-VD-OPh led to a significant reduction of around 45% in the percentage of active caspase-3-positive cells (Fig 3A), as estimated by flow cytometry, but did not completely inhibit the expression of caspase-3 in the retina (Fig 3C and 3D). Interestingly, the entry of microglial cells into the retina in the same Q-VD-OPh-treated explants was also significantly reduced by around 35% (Fig 3B). Hence, the reduction caused by Q-VD-OPh in the percentage of active caspase-3-positive cells and microglial cells entering the retina strongly suggested a relationship between retinal cell death and the entry of microglial cells into the retina.

### Inhibition of purinergic signaling in E6.5+24hiv retina explants blocks the entry of microglial cells into the retina

Extracellular nucleotides such as ATP/ADP and UTP/UDP are known to act as “find-me” and “eat-me” signals that are released by apoptotic cells and attract microglia and other phagocytes [30, 34–37, 52]. Given that the beginning of microglial cell entry into the E7 retina coincided with an increase in active caspase-3-positive apoptotic cells (Figs 1 and 2), it is reasonable to hypothesize that purinergic signaling by nucleotides released from dying cells may play a role in this entry. This hypothesis was tested by treating E6.5 retina explants with either the nucleotide-hydrolyzing enzyme apyrase or the purinergic receptor antagonist suramin.

**Fig 4:**
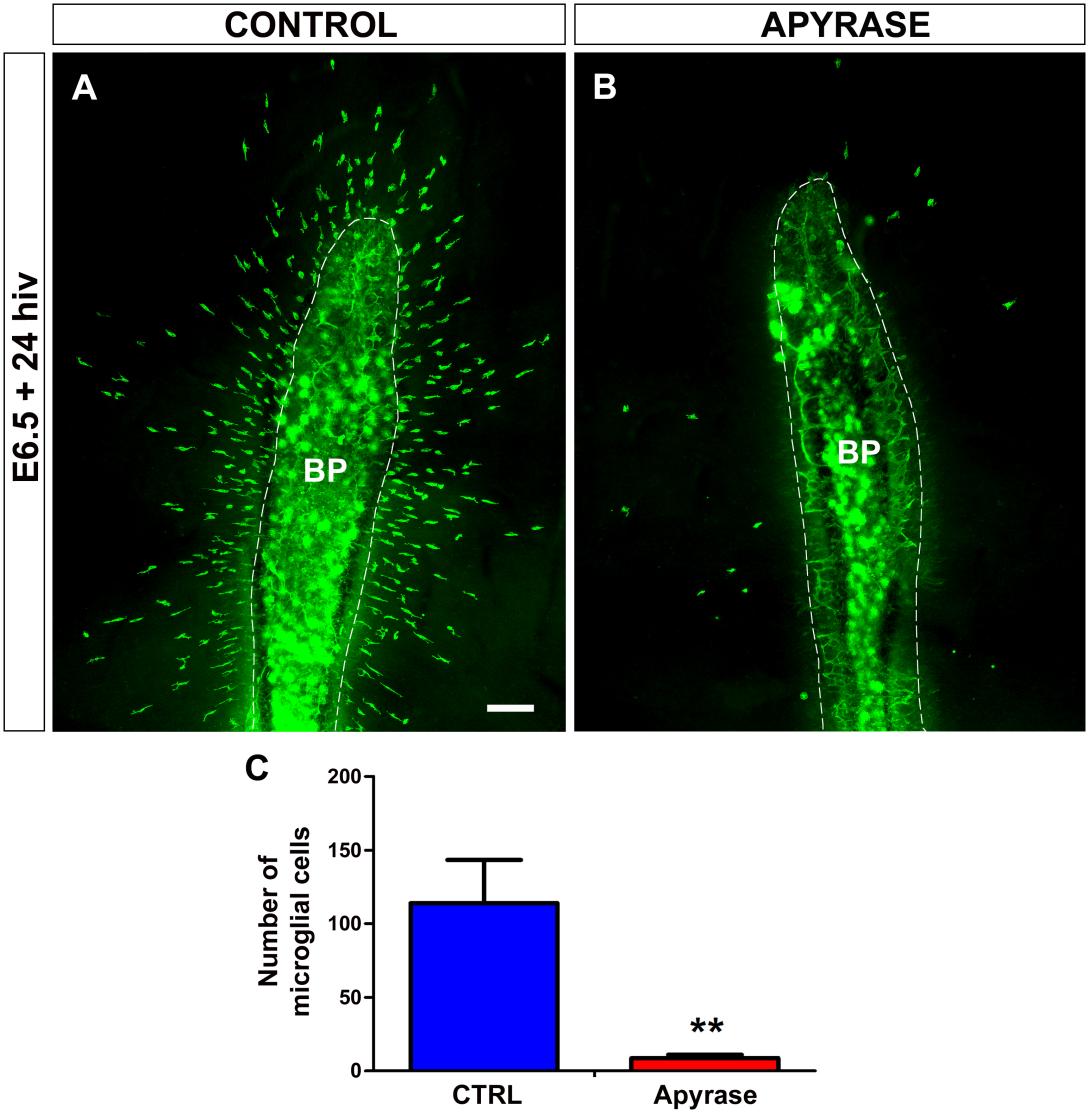
*In vitro* treatment with apyrase inhibits the entry of microglial cells into the embryonic quail retina. **(A, B)** QH1-immunolabeled (green) whole-mounted retina explants from a quail embryo at 6.5 days of incubation cultured for 24 hours *in vitro* (E6.5+24hiv) in apyrase-free (CONTROL, **A**) or 10 U/ml apyrase-containing (APYRASE, **B**) medium. Considerable numbers of QH1-positive microglial cells have entered the retina from the base of the pecten (BP, delimited with a dashed line) in the non-treated control explant (**A**), whereas very few microglial cells are seen in the apyrase-treated explant (**B**). Scale bar: 100 μm. (**C)** Number of microglial cells migrating within the retina is significantly lower in apyrase-treated (Apyrase, red bar) than in non-treated (CTRL, blue bar) E6.5+24hiv retina explants. Data are expressed as means ± SEM (n = 10). Asterisks depict significant differences (**P<0.01, Student´s t-test).

**Fig 5:**
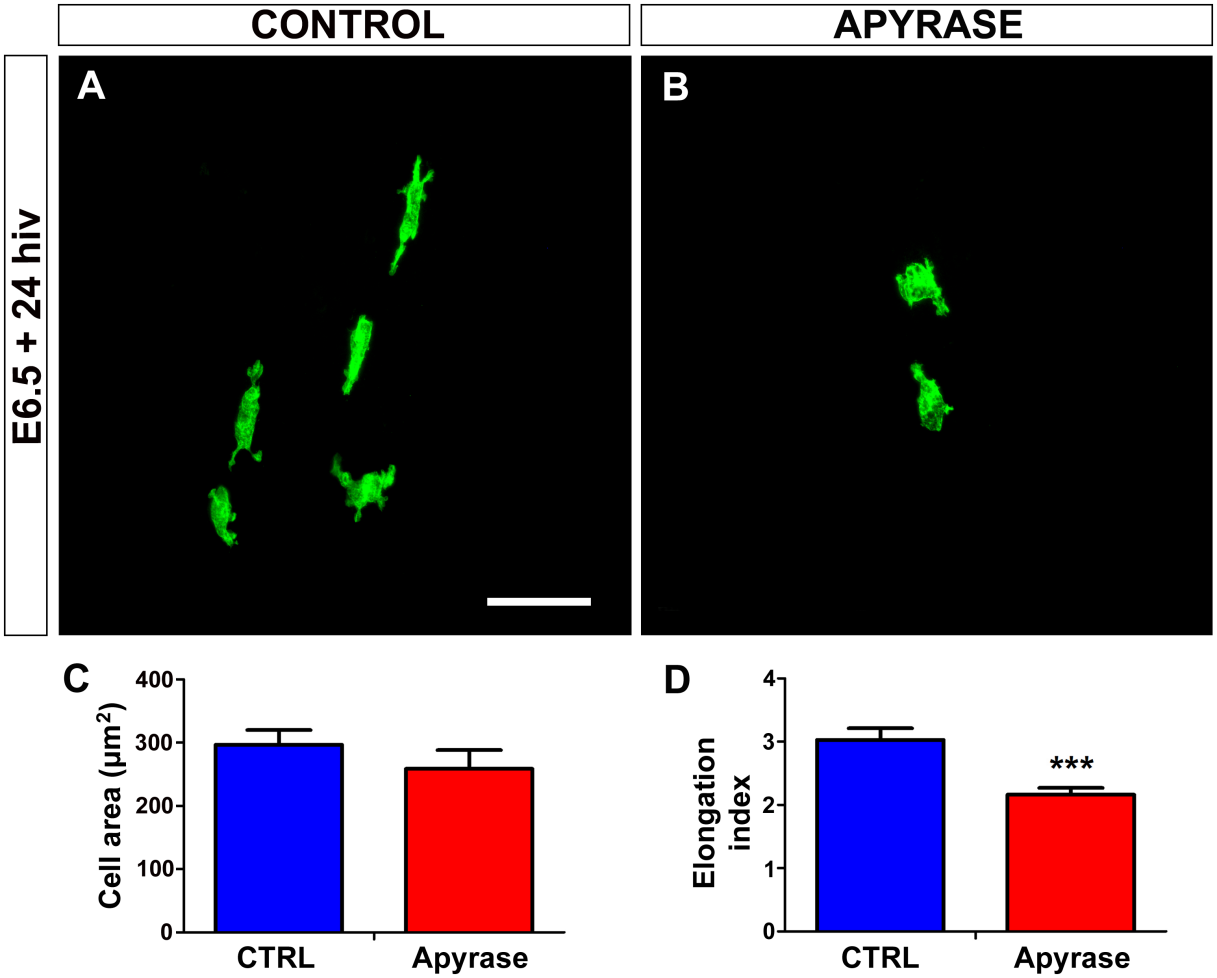
*In vitro* treatment with apyrase induces rounding of microglial cells in quail embryo retina explants. **(A, B)** QH1-positive microglial cells (green) in retina explants from a quail embryo at 6.5 days of incubation cultured for 24 hours *in vitro* (E6.5+24hiv) in apyrase-free (CONTROL, **A**) or apyrase-containing (APYRASE, **B**) medium. Microglial cells in the apyrase-treated explant are more rounded and less elongated (**B**) than those in the control explant, where they show the typical morphology of cells migrating tangentially in the retina, with an elongated cell body and polarized processes (**A**). Scale bar: 50 μm. **(C, D)** Morphometric analysis of cell area (**C**) and elongation index (**D**) for microglial cells in non-treated (CTRL, blue bars) and apyrase-treated (Apyrase, red bars) E6.5+24hiv retina explants. Data are expressed as means ± SEM (n = 24). Asterisks depict significant differences (***P<0.001, Student´s t-test). Although the cell area does not significantly differ between apyrase-treated and non-treated explants (**C**), the elongation index is significantly lower in apyrase-treated explants than in non-treated controls (**D**).

Apyrase abrogates both ATP/ADP and UTP/UDP signaling because it hydrolyzes extracellular tri- and di-phosphate nucleotides [37, 53]. Treatment of retina explants for 24 hiv with 10 U/ml apyrase effectively inhibited the entry of microglial cells into the retina (Fig 4), indicating that tri- and di-phosphate nucleotides but not adenosine were involved in attracting microglial cells from the BP/ONH area. Importantly, the scarce microglial cells entering the retina in apyrase-treated explants had a significantly less elongated phenotype in comparison to the non-treated controls (Fig 5), suggesting that their migratory capacity was limited by the extracellular nucleotide loss.

**Fig 6:**
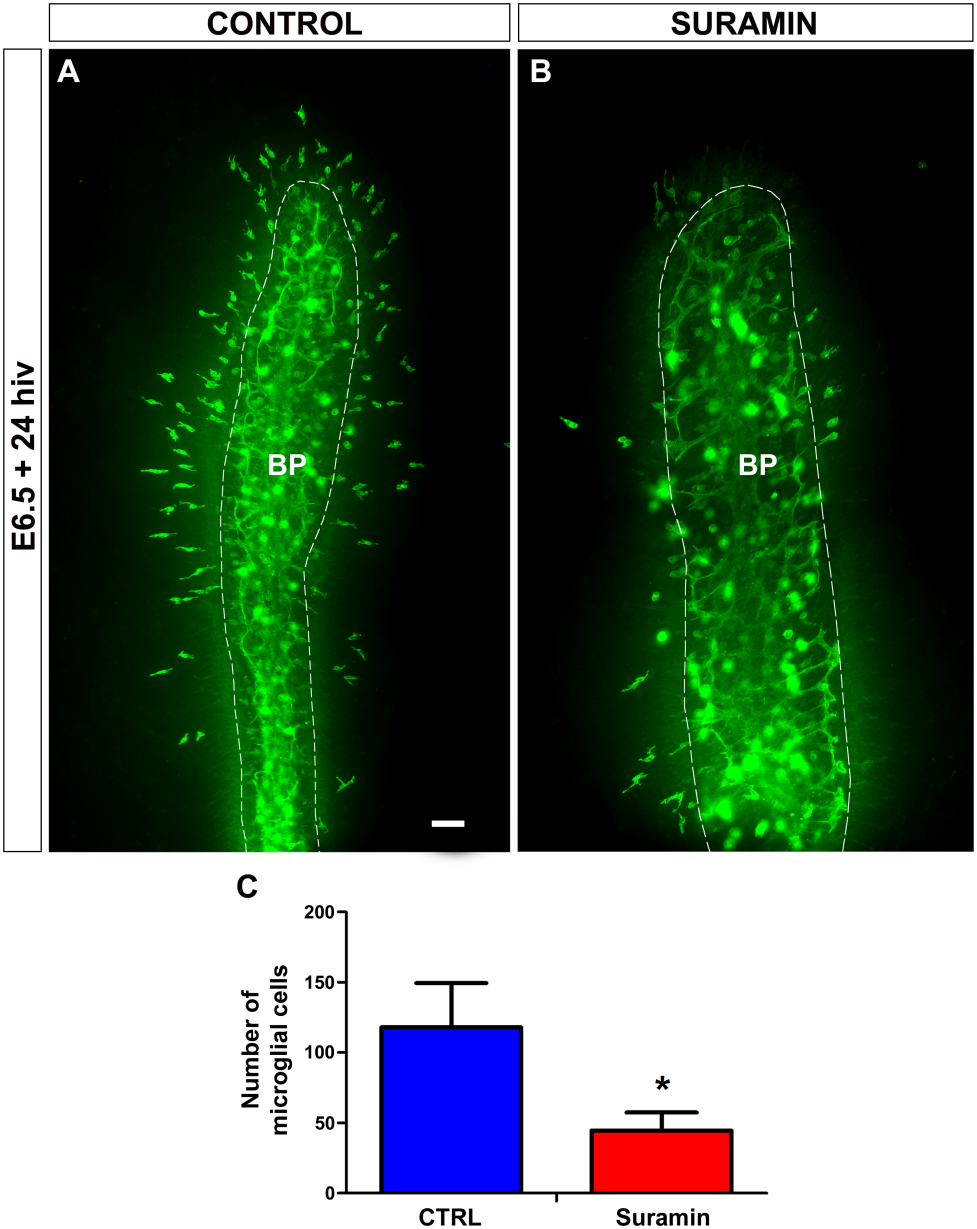
*In vitro* treatment with suramin inhibits entry of microglial cells into the embryonic quail retina. **(A, B)** QH1-immunolabeled (green) whole-mounted retina explants from a quail embryo at 6.5 days of incubation cultured for 24 hours *in vitro* (E6.5+24hiv) in suramin-free (CONTROL, **A**) or 100 μM suramin-containing (SURAMIN, **B**) medium. Abundant QH1-positive microglial cells have entered the retina from the base of the pecten (BP, delimited with a dashed line) in the non-treated control explant (**A**), whereas very scarce microglial cells are seen in the suramin-treated explant (**B**). Scale bar: 100 μm **(C)**. Number of microglial cells migrating within the retina is significantly lower in suramin-treated E6.5+24hiv retina explants (Suramin, red bar) than in non-treated controls (CTRL, blue bar). Data are expressed as means ± SEM (n = 15). Asterisk depicts significant differences (*P<0.05, Student´s t-test).

**Fig 7:**
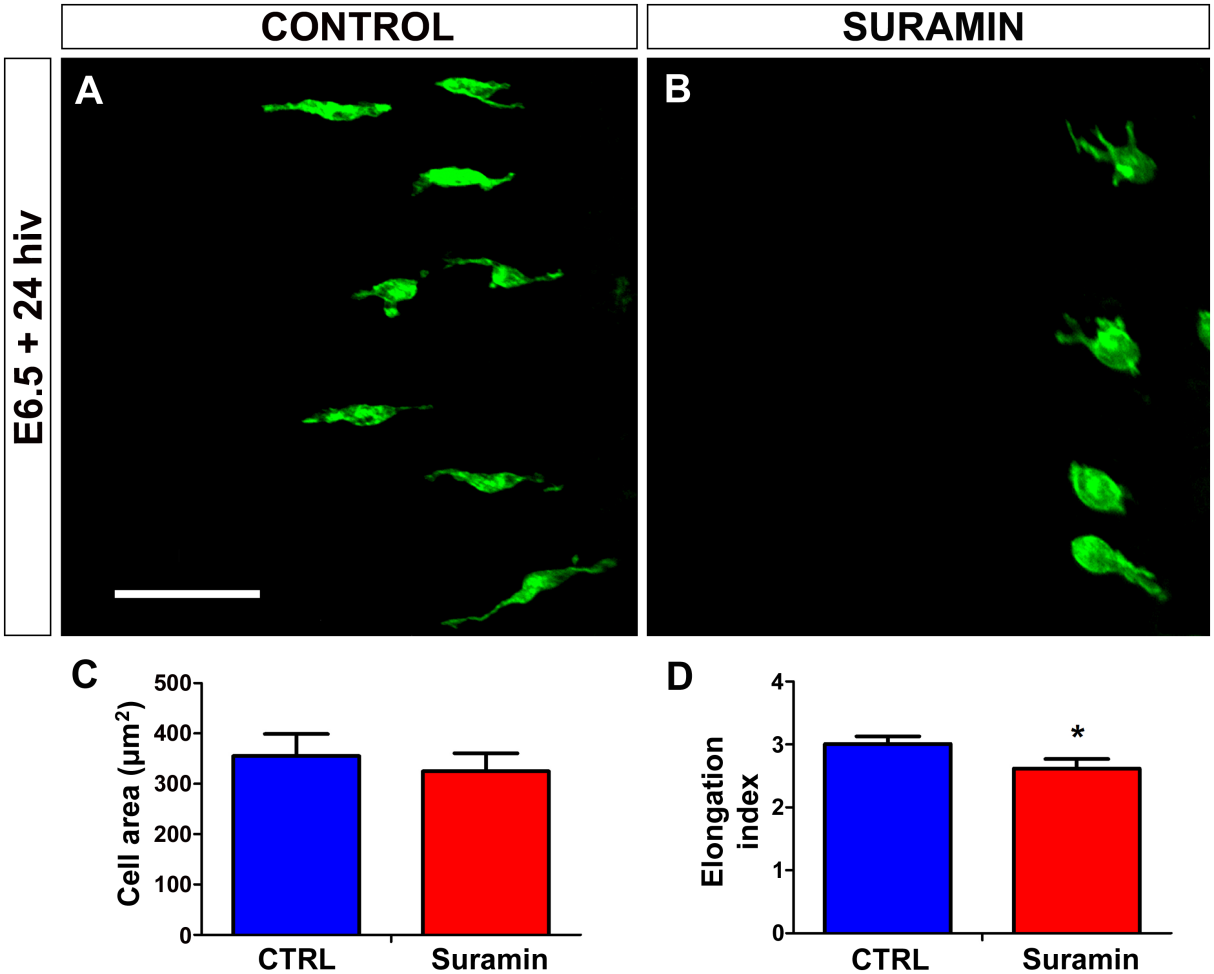
*In vitro* treatment with suramin induces rounding of microglial cells in quail embryo retina explants. **(A, B)** QH1-positive microglial cells (green) in retina explants from a quail embryo at 6.5 days of incubation cultured for 24 hours *in vitro* (E6.5+24hiv) in suramin-free (CONTROL, **A**) or suramin-containing (SURAMIN, **B**) medium. Microglial cells are more rounded and less elongated in the suramin-treated explant (**B**) than in the control explant, where they show the typical polarized morphology of cells migrating tangentially in the retina (**A**). Scale bar: 100 μm. **(C, D)** Morphometric analysis of cell area (**C**) and elongation index (**D**) for microglial cells in non-treated (CTRL, blue bars) and suramin-treated (Suramin, red bars) E6.5+24hiv retina explants. Data are expressed as means ± SEM (n = 45). Asterisk depicts significant differences (*P<0.05, Student´s t-test). The cell area does not significantly differ between suramin-treated and non-treated explants (**C**), but the elongation index is significantly lower in suramin-treated explants than in non-treated controls (**D**).

It is well known that microglia use ionotropic P2X and metabotropic P2Y purinergic receptors to respond to extracellular nucleotides such as ATP/ADP and UTP/UDP [40–43]. In order to verify that P2X/P2Y purinergic receptors were involved in the early entry and migration of microglial cells into the retina, E6.5 retina explants were treated for 24 hiv with the nonspecific P2X/P2Y receptor antagonist suramin [54–56], finding that treatment with 100 μM suramin significantly prevented the entry of microglial cells into the retina (Fig 6). Moreover, as observed with the apyrase treatment, the very few microglial cells found within the retina of suramin-treated explants had a significantly less elongated phenotype in comparison to non-treated controls (Fig 7), suggesting that these cells ceased tangential migration after their purinergic receptors were blocked.

**Fig 8:**
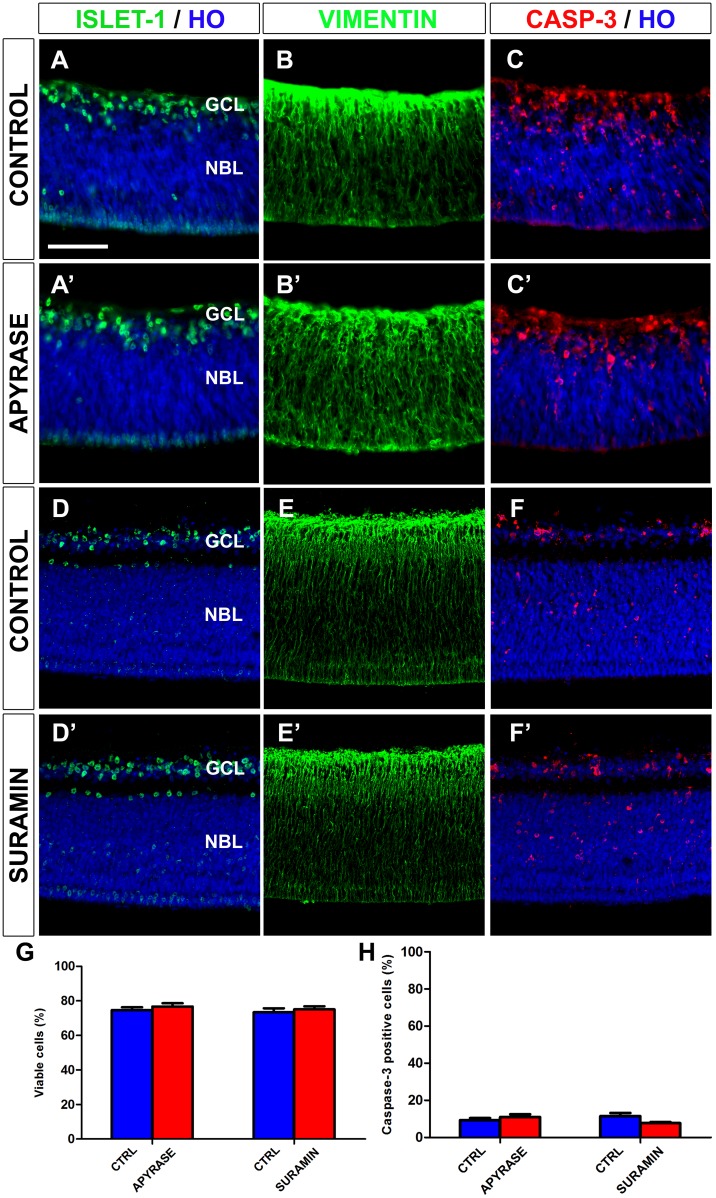
*In vitro* treatment with apyrase or suramin does not alter cell viability or the distribution patterns of active caspase-3-, islet-1-, or vimentin-positive cells in quail embryo retina explants. Single anti-islet-1 (**A, A’, D, D’**), anti-vimentin (**B, B’, E, E’**), and anti-active caspase-3 (**C, C’, F, F’**) immunostained cross-sections of retina explants from quail embryos at 6,5 days of incubation cultured for 24 hours *in vitro* in the presence of apyrase (APYRASE, **A’**, **B’, C’**) or suramin (SURAMIN, **D’**, **E’, F’**). Cross sections of apyrase-free and suramin-free explants (CONTROL) are shown in **A, B, C** and **D, E, F**, respectively. Cell nuclei are stained with Hoechst 33342 (HO, blue). Islet-1-positive neurons are mainly located in the ganglion cell layer (GCL). Vimentin-positive cells are distributed throughout the retina thickness, with higher expression in the GCL. Caspase-3-positive dying cells are mainly located in the GCL and the inner half of the neuroblastic layer (NBL). No changes in retina cytoarchitecture or caspase-3 expression pattern are observed between apyrase- or suramin-treated explants and their respective non-treated controls. Scale bar: 50 μm. (**G, H**) Percentages of viable cells (**G**) and caspase-3-positive cells (**H**), as determined by flow cytometry, in apyrase- and suramin-treated explants (red bars) and their respective non-treated controls (blue bars). Data are expressed as means ± SEM (n = 6 for each). No significant differences in cell viability or caspase-3-positive cell percentage are observed between explants treated with apyrase or suramin and their respective non-treated controls.

Taken together, the results of our experiments with apyrase and suramin treatment showed that P2X/P2Y purinergic signaling plays a key role in the early entry and tangential migration of microglial cells in the E7 quail embryo retina. We highlight the fact that neither apyrase nor suramin modified the expression pattern of active caspase-3, the percentage of active caspase-3 positive cells in the retina, or the retinal cell cytoarchitecture in cultured explants (Fig 8). Thus, the apyrase- or suramin-treated E6.5+24hiv retina explants and their respective controls all showed similar caspase-3 expression patterns in the GCL and the neuroblastic layer, the only two cell layers observed in the retina at this stage of development (compare Fig 8C and 8F with Fig 8C’ and 8F’, respectively), and similar percentages of active caspase-3-positive cells in the retina (Fig 8H). The presence of active caspase-3-positive cells was particularly high in the incipient GCL and the inner half of the neuroblastic layer of cultured retina explants (Fig 8C, 8C’, 8F and 8F’), as also observed in the *in situ* retina (Fig 1). In addition, the percentage of viable cells (Fig 8G) and the distribution pattern of cell markers, such as vimentin (cytoskeletal protein specific to Müller cells) or the transcription factor islet-1 (expressed by ganglion cells and other subsets of retinal neurons), did not differ between apyrase- or suramin-treated retina explants and their respective controls (compare Fig 8A, 8B, 8D and 8E with Fig 8A’, 8B’, 8D’ and 8E’, respectively). These results suggest that the effects of apyrase or suramin treatments on microglial cells were not due to an indirect effect on other retinal cell types.

### Exogenous ATP and UDP induce an increase in the number of microglial cells entering the retina

After establishing the contribution of purinergic signaling to the initiation of microglial cell colonization of the quail retina, we investigated the involvement of different extracellular nucleotides in this signaling. ATP and UDP, known to act as signals released by damaged neurons that stimulate chemotaxis and chemokinesis of microglial cells [34–37, 39, 53], were selected for testing, treating E6.5 retina explants with either nucleotide for 24 hiv.

**Fig 9:**
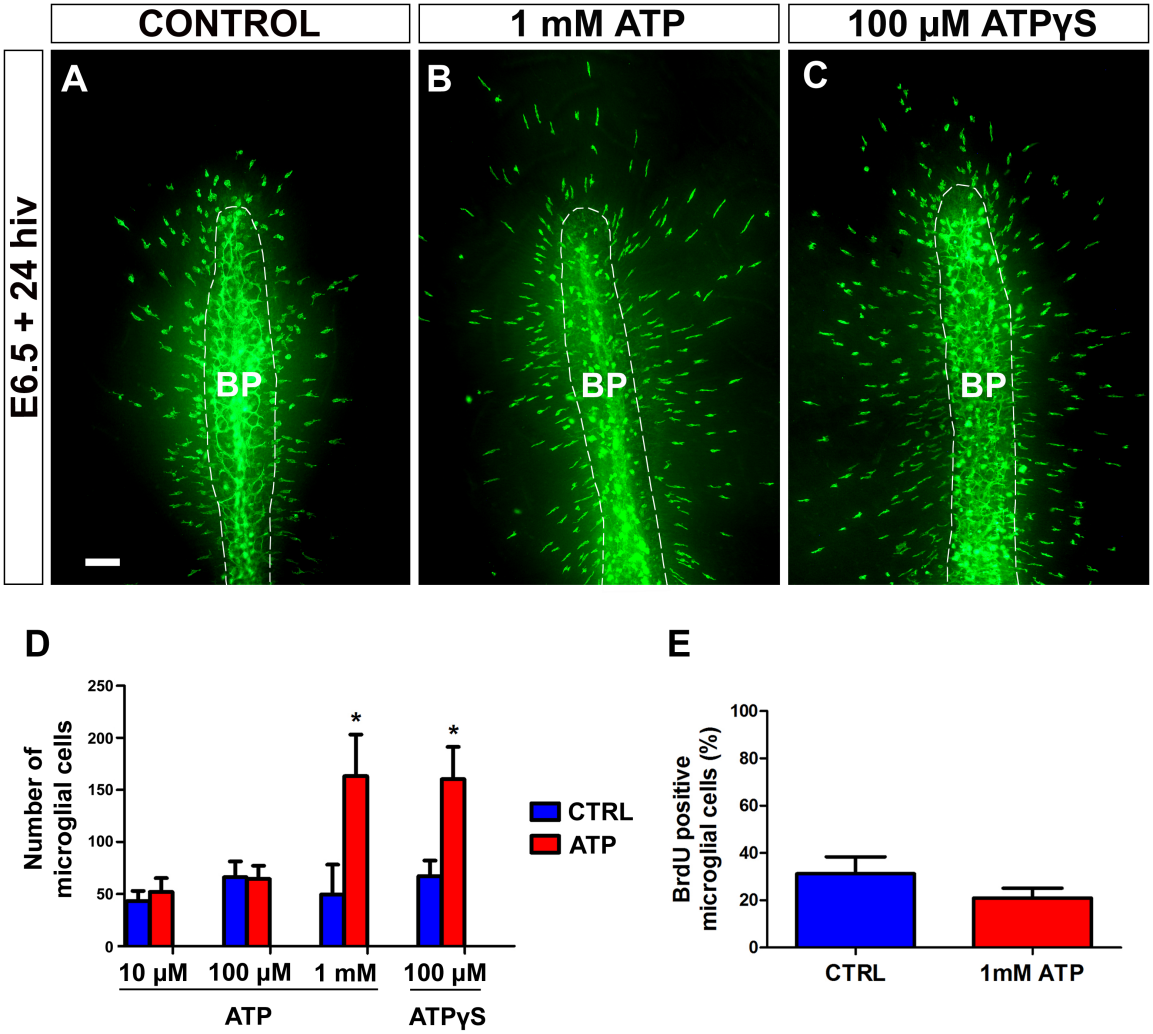
*In vitro* treatment with 1 mM ATP or non-hidrolyzable ATP (ATPγS) stimulates entry of microglial cells into the embryonic quail retina. **(A-C)** QH1-immunolabeled (green) whole-mounted retina explants from quail embryos at 6.5 days of incubation cultured for 24 hours *in vitro* (E6.5+24hiv) in ATP-free medium (CONTROL, **A**) or in medium containing 1mM ATP (**B**) or 100 μM ATPγS (**C**). QH1-labeled microglial cells appear to be more abundant in ATP- and ATPγS-treated retina explants than in the non-treated explant. BP: base of the pecten (delimited with a dashed line). Scale bar: 100 μm. (**D)** Number of microglial cells migrating within the retina in explants treated with different concentrations of ATP (10 μM, 100 μM, and 1 mM) or 100 μM ATPγS (red bars) and in their respective non-treated controls (blue bars). Data are expressed as means ± SEM (n = 10 for each). Asterisks depict significant differences (*P<0.05, Student´s t-test). Treatments with 1 mM ATP and 100 μM ATPγS significantly increase the number of microglial cells in E6.5+24hiv retina explants. In contrast, no significant differences are observed between explants treated with low ATP concentrations (10 μM and 100 μM) and their non-treated controls. (**E**) Percentages of BrdU-positive microglial cells in retina explants treated with 1 mM ATP (red bar) and non-treated controls (blue bar). Data are expressed as means ± SEM (n = 8). No significant differences are detected between ATP-treated and non-treated explants.

**Fig 10:**
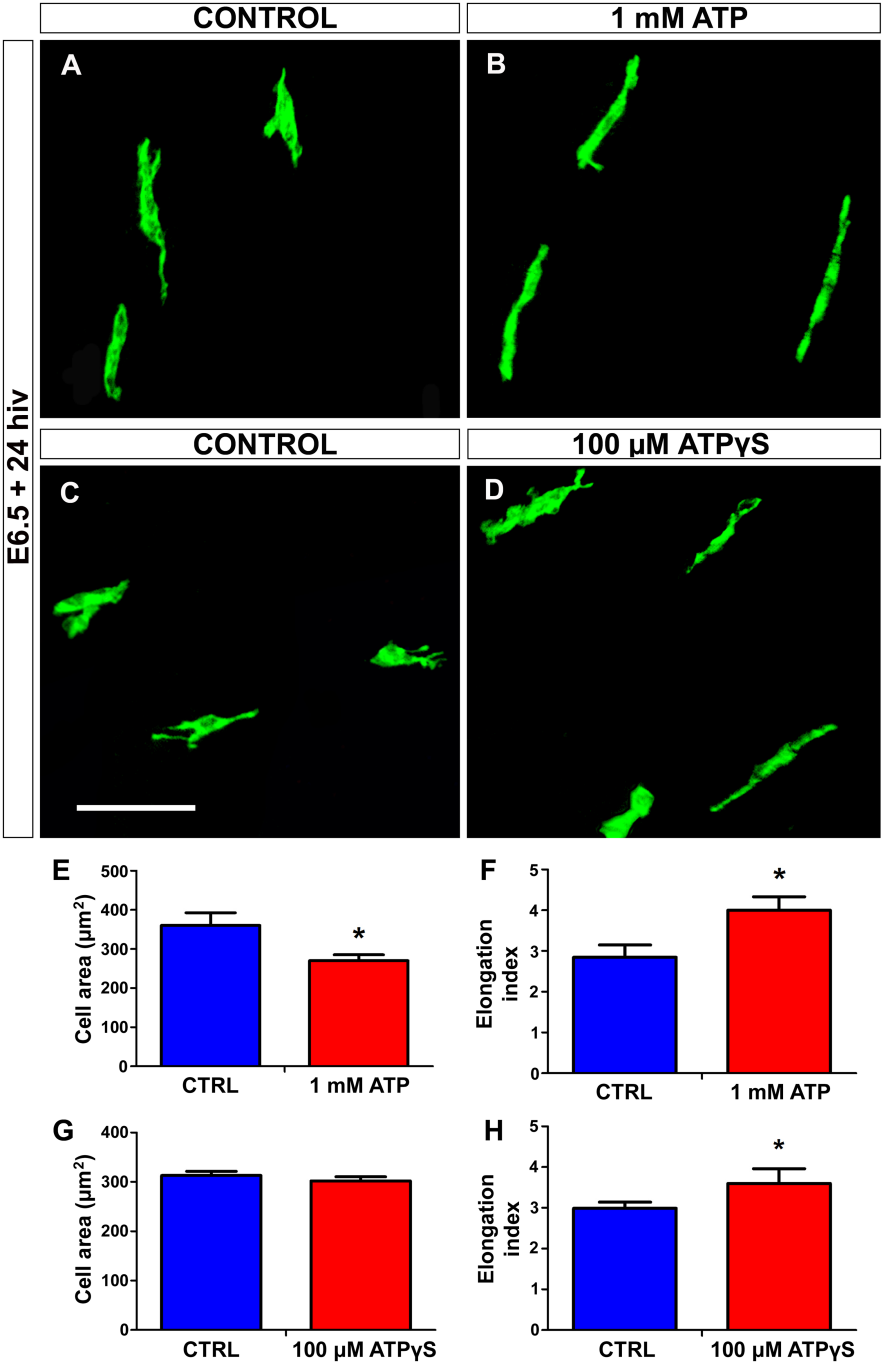
*In vitro* treatment with ATP or non-hydrolyzable ATP (ATPγS) induces elongation of microglial cells in quail embryo retina explants. **(A-D)** QH1-immunolabeled microglial cells (green) in retina explants from quail embryos at 6.5 days of incubation cultured for 24 hours *in vitro* (E6.5+24hiv) in ATP-free (CONTROL, **A**), 1 mM ATP-containing (1 mM ATP, **B**), ATPγS-free (CONTROL, **C**), or 100 μM ATPγS-containing (**D**) medium. Microglial cells appear more elongated in ATP- and ATPγS-treated explants than in their respective control explants, which is compatible with a greater migratory activity of microglial cells in nucleotide-treated explants. Scale bar: 50 μm. **(E-H)** Morphometric analysis of cell area (**E** and **G**) and elongation index (**F** and **H**) for microglial cells in non-treated (CTRL, blue bars), 1 mM ATP-treated (red bars), and 100 μM ATPγS-treated (red bars) E6.5+24hiv retina explants. Data are expressed as means ± SEM (n = 30 for each). Asterisks depict significant differences (*P<0.05, Student´s t-test). The elongation index is significantly higher in 1 mM ATP- and 100 μM ATPγS-treated explants than in their respective non-treated controls.

Exogenous ATP at a concentration of 1 mM, but not of 10 or 100 μM, produced a significant increase in the number of microglial cells entering the retina (Fig 9). The fact that ATP only had a microglia-attracting effect at a high concentration suggests that ATP might be hydrolyzed to AMP or adenosine by the action of ectonucleotidases such as nucleoside triphosphate diphosphohydrolase (NTDPase), nucleoside diphosphatase (NDPase), and/or ecto-5’-nucleotidase (5’-NT), which are known to be expressed by microglial cells and appear to play an important role in ATP-induced microglia guiding [40, 42, 57, 58]. In order to test whether ectonucleotidases acted in our *in vitro* system, retina explants were treated for 24 hiv with the non-hydrolyzable ATP analogue ATPγS. Treatment with 100 μM ATPγS induced a similar increase in the entry of microglial cells into the retina to that observed in explants treated with 1 mM hydrolyzable ATP (Fig 9). The fact that a 10-fold higher concentration of hydrolyzable ATP is needed to produce the same effect as that of 100 μM non-hydrolyzable ATPγS supports the proposition that ATP may be hydrolyzed to AMP or adenosine by the action of ectonucleotidases. It should also be taken into account that a larger number of microglial cells in the retina may result from increases not only in microglial migration but also in microglial proliferation. E6.5+24hiv retina explants treated with 1mM ATP and their respective controls were additionally incubated with BrdU for the last 12 hiv, selecting this time point because microglial cells enter the retina from E6.5+12hiv onward (unpublished results), the *in vitro* time equivalent to E7 in the *in situ* retina. No significant differences were detected in the percentage of BrdU-positive microglial cells between ATP-treated explants and their controls (Fig 9E), showing that exogenous ATP did not affect the proliferative activity of microglial cells. Furthermore, both 1 mM ATP and 100 μM ATPγS caused a significant elongation of microglial cells migrating within the retina (Fig 10), indicating that exogenous extracellular ATP increases the chemokinesis of these cells after their entry into the retina.

**Fig 11:**
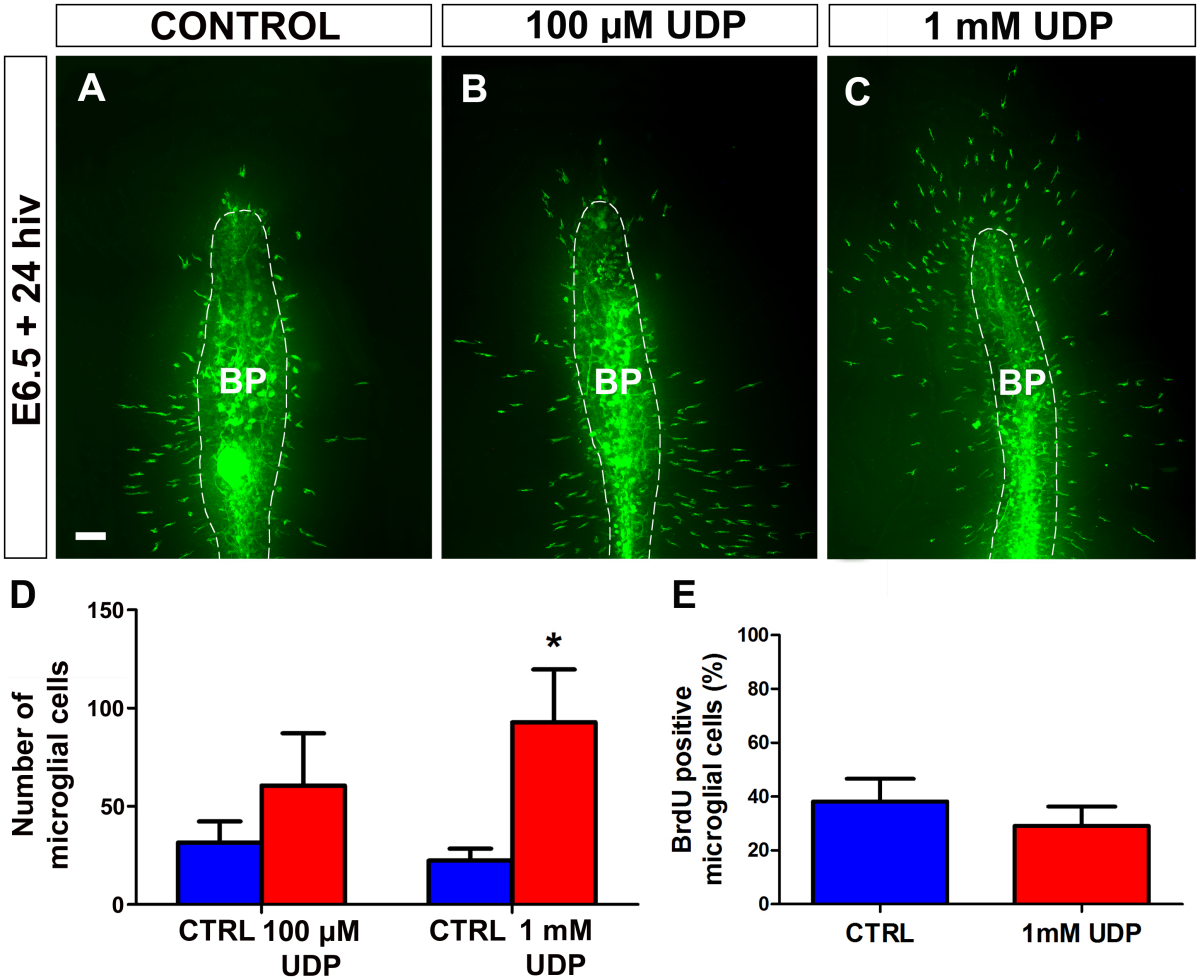
*In vitro* treatment with UDP stimulates entry of microglial cells into the embryonic quail retina. **(A-C)** QH1-immunolabeled (green) whole-mounted retina explants from quail embryos at 6.5 days of incubation cultured for 24 hours *in vitro* (E6.5+24hiv) in UDP-free medium (CONTROL, **A**) or in media containing 100 μM UDP (**B**) or 1 mM UDP (**C**). QH1-labeled microglial cells appear more abundant in both UDP-treated retina explants than in the non-treated explant. BP: base of the pecten (delimited with a dashed line). Scale bar: 100 μm. **(D)** Number of microglial cells migrating within the retina in 100 μM UDP- and 1mM UDP-treated E6.5+24hiv retina explants (red bars) and in their respective non-treated controls (blue bars). Data are expressed as means ± SEM (n = 10 for each). Asterisk depicts a significant difference (*P<0.05, Student´s t-test). The number of microglial cells entering the retina increases after UDP treatment at both concentrations, but the increase only reaches significance at the higher concentration (1 mM). (**E**) Percentages of BrdU-positive microglial cells in retina explants treated with 1 mM UDP (red bar) and non-treated controls (blue bar). Data are expressed as means ± SEM (n = 8). No significant differences are observed between UDP-treated and non-treated explants.

**Fig 12:**
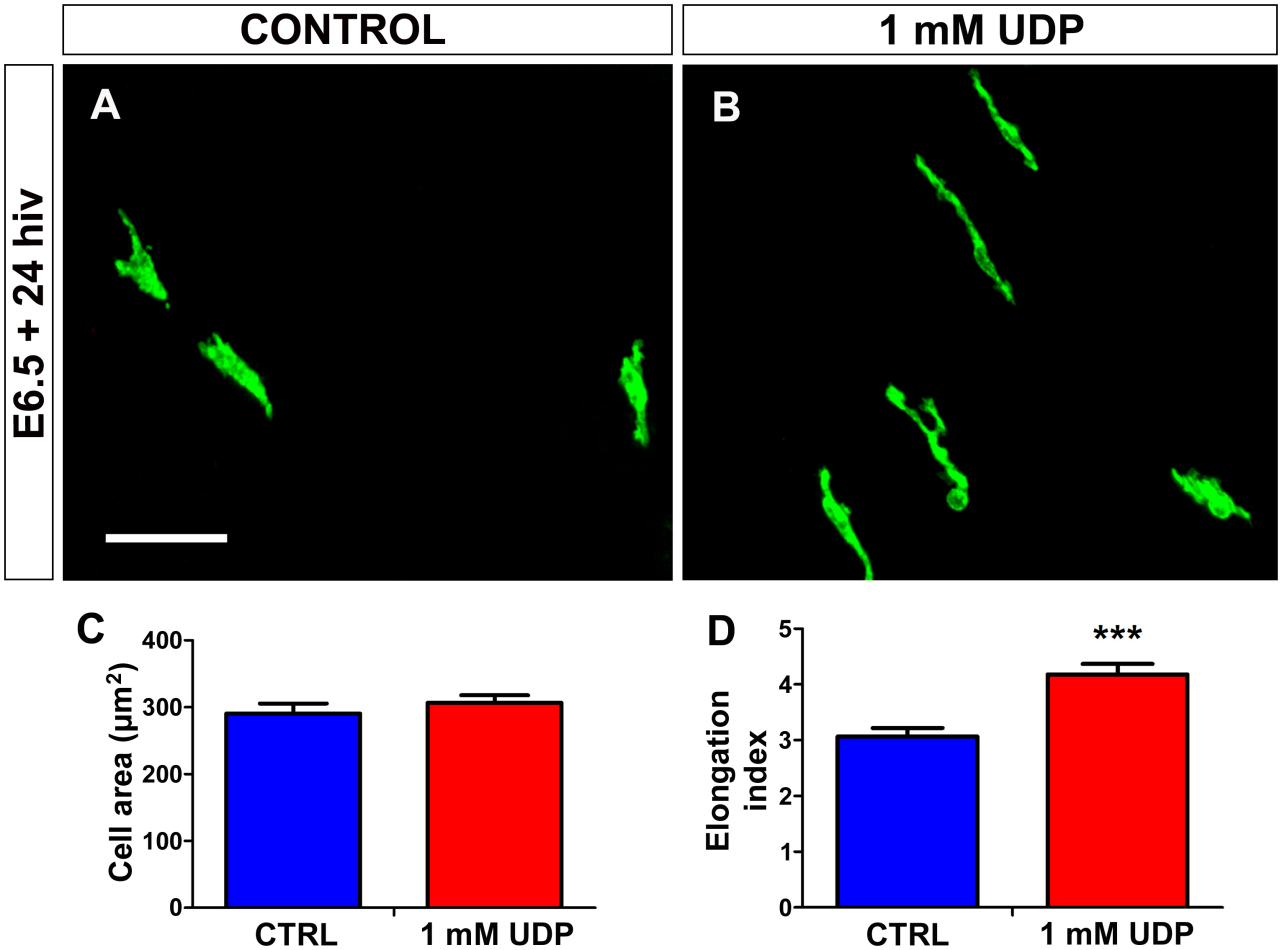
*In vitro* treatment with UDP induces elongation of microglial cells in quail embryo retina explants. **(A, B)** QH1-immunolabeled microglial cells (green) in retina explants from a quail embryo at 6.5 days of incubation cultured for 24 hours *in vitro* (E6.5+24hiv) in UDP-free (CONTROL, **A**) or 1 mM UDP-containing (1 mM UDP, **B**) medium. Microglial cells appear more elongated in UDP-treated explants than in their respective control explants, which is compatible with a greater migratory activity of microglial cells in UDP-treated explants. Scale bar: 50 μm. **(C, D)** Morphometric analysis of cell area (**C**) and elongation index (**D**) for microglial cells in non-treated (CTRL, blue bars) and 1 mM UDP-treated (red bars) E6.5+24hiv retina explants. Data are expressed as means ± SEM (n = 30). Asterisks depict significant differences (***P<0.001, Student´s t-test). The elongation index is significantly higher in UDP-treated explants than in non-treated controls.

Treatment of E6.5 retina explants with exogenous UDP for 24 hiv also produced an increase in the number of microglial cells entering the retina (Fig 11), which was less pronounced with 100 μM UDP but reached statistical significance with 1 mM UDP (Fig 11D). Treatment with 1 mM UDP did not significantly affect the proliferation of microglial cells that had entered the retina (Fig 11E) and caused a significant increase in cell microglial elongation (Fig 12), similar to that observed after ATP treatment. The similarity of the effects produced by treatment with ATP and UDP on the entry and elongation of microglial cells suggested that both nucleotides might act on the same receptors, probably metabotropic P2Y receptors, which are the purinergic receptors signalized by both ATP and UDP.

**Fig 13:**
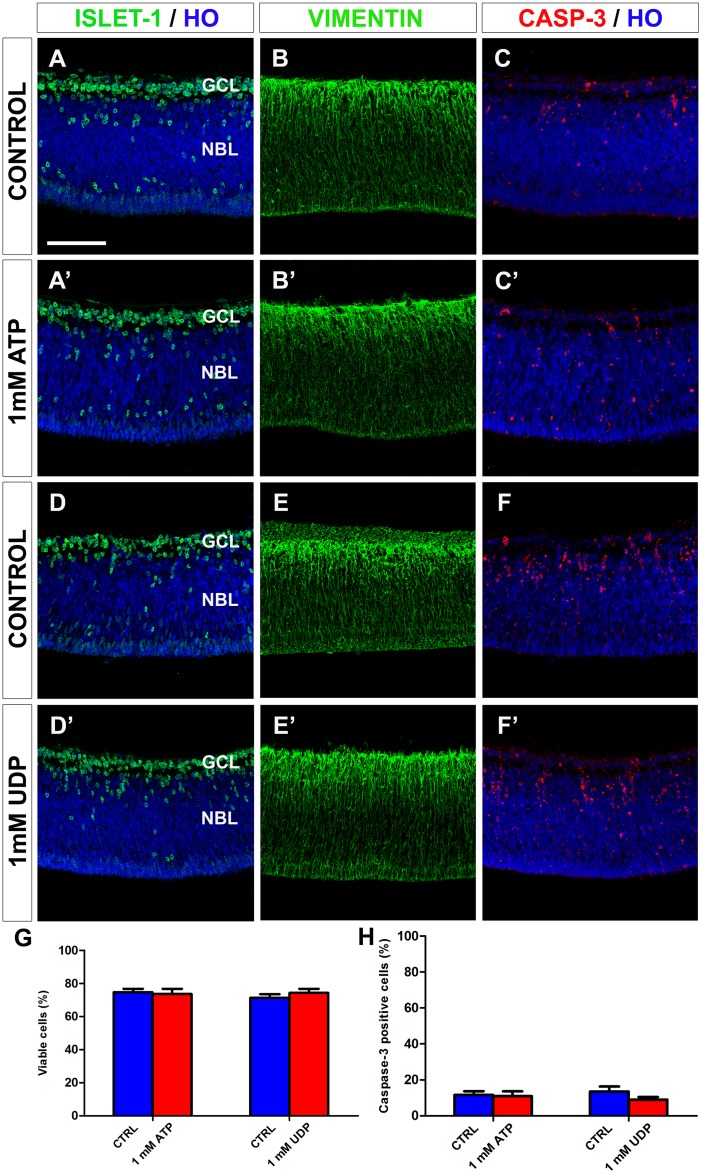
*In vitro* treatment with ATP or UDP does not alter cell viability or the distribution patterns of active caspase-3-, islet-1-, or vimentin-positive cells in quail embryo retina explants. (**A-F**) Single anti-islet-1 (**A, A’, D, D’**), anti-vimentin (**B, B’, E, E’**) and anti-active caspase-3 (**C, C’, F, F’**) immunostained cross-sections of retina explants from quail embryos at 6.5 days of incubation cultured for 24 hours *in vitro* in the presence of 1 mM ATP (**A’**, **B’, C’**) or 1 mM UDP (**D’**, **E’, F’**). Cross sections of ATP-free and UDP-free explants (CONTROL) are displayed in **A, B, C** and **D, E, F**, respectively. Cell nuclei are stained with Hoechst 33342 (HO, blue). Islet-1-positive neurons are mainly located in the ganglion cell layer (GCL). Vimentin-positive cells are distributed throughout the retina thickness, with higher expression in the GCL. Caspase-3-positive dying cells are mainly located in the GCL and the inner half of the neuroblastic layer (NBL). The retina cytoarchitecture and caspase-3 expression pattern remain unaltered after ATP or UDP treatment. Scale bar: 50 μm. (**G, H**) Percentages of viable cells (**G**) and caspase-3-positive cells (**H**), as determined by flow cytometry, in ATP- or UDP-treated explants (red bars) and their respective non-treated controls (blue bars). Data are expressed as means ± SEM (n = 6 for each). No significant differences in cell viability or caspase-3-positive cell percentage are observed between explants treated with 1 mM ATP or 1 mM UDP and their respective non-treated controls.

The effects of exogenous ATP and UDP on microglial cells were not due to an indirect effect on other retinal cell types, because ATP and UDP treatments did not modify the expression pattern of active caspase-3, the percentages of active caspase-3-positive cells or viable cells, or the distribution pattern of other cell markers such as vimentin and islet-1 (Fig 13), as also found for the apyrase and suramin treatments.

Taken together, the above results reveal that extracellular ATP and UDP increased the early entry and chemokinesis of microglial cells in the quail embryonic retina without affecting their proliferation.

## Discussion

Our group previously showed that microglial cells in the quail retina derive from precursors of amoeboid phenotype that start to enter the embryonic retina from the BP/ONH area at E7 and then migrate tangentially in a central-to-peripheral direction by a similar cellular mechanism to that of cultured fibroblasts *in vitro* [15–17]. In the present study, we demonstrate a spatiotemporal coincidence between the first entry of microglial cells into the developing quail retina and a marked increase in PCD in retinal regions located dorsal to the BP/ONH area. Numerous studies have addressed temporal and spatial relationships between the spread of microglial cells and PCD in various regions of the normal developing CNS, including the retina [16, 21, 22, 25–27, 59–62]. A close physical association between dying neurons and microglial cells is also well documented, with reports that microglia phagocytose neuronal debris [16, 21, 28, 59–61] and even promote the execution of death programs in contacted cells [24, 63–65]. Many of these studies proposed that naturally occurring cell death might attract microglial cells into the developing CNS; however, it was only recently demonstrated for the first time, by two independent research groups, that neuronal apoptosis attracts committed microglial precursors into the developing CNS of the zebrafish [44, 45]. Both studies elegantly showed that the time window and location of microglial colonization of the developing zebrafish brain were positively correlated with the spatiotemporal pattern of neuronal death. By contrast, microglial colonization in the developing hippocampus of postnatal BAX null mice was found to be independent of developmental cell death [59]. These knockout mice showed the reduction but not elimination of cell death in the developing hippocampus, among other brain regions [66]. There was a transient reduction in microglial density in the hippocampus of BAX null mice during the first postnatal week, coinciding with the reduced cell death in these mice, although this density become similar to that in wild-type mice during the second postnatal week [59]. In our view, these findings support a close relationship between early microglial colonization and developmental cell death, although factors other than neuronal death would participate in regulating later microglial recruitment into the hippocampus, as proposed by the authors [59]. The present observations show a clear spatial and temporal correlation between the first entry of microglial precursors into the quail embryo retina at E7 and increased cell death in the central retina, as detected by the expression of active caspase-3 and TUNEL staining (see Fig 1). In addition, we found that inhibition of caspases in E6.5+24hiv retina explants significantly reduces active caspase-3 expression in parallel with a significant decrease in the entry of microglial cells into the retina (Fig 3). These results suggest a link between microglial recruitment and PCD and are in agreement with the findings of the above-cited studies in the developing zebrafish brain [44, 45]. This hypothesis is also supported by the frequent observation of contact between microglial precursors entering the retina and the axons and somas of active caspase-3-positive ganglion cells (Fig 1).

After establishing that the early entry of microglial precursors into the developing quail retina could be triggered by PCD, we investigated the potential molecular mechanisms involved in this process, focusing on purinergic signaling. Extracellular nucleotides such as ATP/ADP and UTP/UDP have been extensively described in diseased or damaged CNS tissues as molecules released by dying neurons, inducing migration and/or phagocytosis in microglial cells via different P2X and P2Y purinergic receptors on their surface [41–43]. Thus, extracellular ATP leaked from injured cells has been found to exert both chemotactic and chemokinetic effects on microglial cells by binding to P2X_4_, P2Y_1_ and P2Y_12_ purinergic receptors [34, 35, 37, 39, 67–69]. Injured cells also release UTP that is immediately degraded by extracellular ectonucleotidases to UDP, which acts on microglial P2Y_6_ receptors to facilitate phagocytosis [36, 53]. However, the role of these nucleotides and their receptors in the entry and migration of microglial cells during normal development has not yet been elucidated. Notably, nucleotide signaling was recently shown to participate in the entry of microglial precursors into the developing zebrafish brain, although there was no investigation into the nature of the nucleotides involved or into the cellular mechanisms that underlie this entry [44].

The role of purinergic signaling and extracellular nucleotides in the entry of microglial precursors into the retina was analyzed in this study by using organotypic cultures of E6.5 quail embryo retina explants, mimicking the first entry and migration of microglial precursors in the *in situ* developing retina. We ascertained that around 20 to 150 microglial cells entered the retina per explant and that a similar number was always found in explants from both eyes of each embryo. The *in vitro* culture of retina explants started 12 hours before the first entry of microglial precursors into the retina and stopped 12 hours later, a narrow window of development that allowed the study of factors controlling their initial entry and migration into the retina in a highly similar state to that of the *in situ* retina.

Purinergic signaling was blocked in E6.5 retina explants by using two complementary approaches. In the first, apyrase, which hydrolyzes extracellular tri- and di-phosphate nucleotides [37, 70], was added to the culture medium, thereby eliminating both ATP/ADP and UTP/UDP signaling. In the second, E6.5 retina explants were treated with the nonspecific P2X/P2Y receptor antagonist suramin [54, 55]. Both treatments effectively inhibited the entry of microglial cells into the retina, demonstrating that: i) tri- and di-phosphate nucleotides are involved in the migration of microglial cells from the BP/ONH area, and ii) P2X/P2Y purinergic signaling plays an essential role in the first entry and migration of microglial cells in the E7 quail embryo retina. These results are in agreement with the report by Casano et al. [44] that entry of microglial precursors into the zebrafish brain is significantly reduced by treating the embryos with suramin or by blocking pannexin-1 channels, which are involved in the extracellular release of cytosolic nucleotides during apoptosis [71, 72]. It is noteworthy that none of the present treatments changed the distribution of active caspase-3-positive cells, the percentages of active caspase-3-positive cells or viable cells in the explants, or the cell cytoarchitecture of the retina. This indicates that the blockade of microglial cell entry into the retina after treatment with apyrase or suramin was not due to an indirect effect on other retinal cells as a consequence of their non-specific toxicity. In addition, the very few microglial cells entering the retina in apyrase- or suramin-treated explants had a significantly less elongated phenotype in comparison to non-treated controls, indicating a limitation in their migratory capacity. These results suggest that purinergic signaling is necessary not only for the first entry of microglial cells into the retina but also for their initial tangential migration, although we cannot rule out additional factors that might regulate this early migration of microglial precursors.

ATP and UDP were added to the culture medium of E6.5 retina explants to verify the involvement of tri- and di-phosphate nucleotides in the migration of microglial precursors toward the retina. Interestingly, treatment with either 1 mM ATP or 1 mM UDP produced a significant and similar increase in the number and elongation of microglial cells in the retina without affecting their proliferation, strongly suggesting that both nucleotides are involved not only in the entry of microglial precursors into the retina from the BP/ONH area but also in their subsequent tangential migration. Given that ATP is able to bind to both P2X and P2Y receptors and UDP can only bind to P2Y receptors [56, 73], we speculate that P2Y purinergic receptors might be the most suitable candidates for participation in these processes, although further investigation is required to test this proposition. In addition, the positive effect of exogenous ATP and UDP on the entry of microglial cells into the retina in our experimental system is compatible with an increased chemokinesis of these cells. It is well known that three forms of cell migration can be distinguished in relation to chemical signals involved in its regulation: (i) basal random locomotion, which takes place in the absence of external chemical stimuli; (ii) chemokinesis, which occurs when a chemical factor stimulates cell migration without determining the direction of migration, i.e., the chemical stimulus increases the random locomotion of cells; and (iii) chemotaxis, which takes place when a soluble factor has a concentration gradient that acts asymmetrically to dictate the direction of cell migration from lower to higher concentration levels [74, 75]. In fact, retina explants were cultured on Millicell membranes vitreal surface down and ATP or UDP were dissolved into the culture medium below membranes. Therefore, nucleotide concentrations would be similar throughout the vitreal surface of the retina. In addition, it has been reported that exogenous ATP or UDP can disrupt possible gradients of these nucleotides released by endogenous apoptotic cells [33, 76]. Thus, our results would be compatible with the existence of a chemokinetic effect of ATP or UDP on microglial cells entering the quail retina at E7. This hypothesis is in accordance with findings in primary cultures of mouse microglial cells that the migratory response of microglia to extracellular ATP may be driven by both chemotaxis and chemokinesis [35, 39].

We highlight that our experiments do not demonstrate the release of ATP or UDP by apoptotic cells in the embryonic quail retina, and we cannot rule out other sources of these nucleotides. ATP and other nucleotides are known to be released by neurons or macroglial cells by various mechanisms, acting as glio- and neurotransmitters in the retina and other CNS regions, and to be involved in mediating bidirectional neuron-glia signaling in developmental and disease processes [77–79]. Light stimulation of the retina was found to increase extracellular levels of ATP, which was released in a calcium-dependent manner from amacrine and/or ganglion cells [80–82]. In common with other conventional neurotransmitters, ATP is stored and released from secretory vesicles in the presynaptic terminal of neurons [83]. It is believed to act as a co-transmitter in many cells, where it is released with both inhibitory (GABA) and excitatory (glutamate) neurotransmitters and neuropeptides [83–86]. Nevertheless, the role of ATP as a neurotransmitter in the developing retina has not been addressed, and further studies are required to establish whether ATP is released by neurons in E6.5 retina explants. In addition, non-neuronal cells are also a source of ATP in the retina, and it has been reported that retinal astrocytes, Müller cells, and retinal pigment epithelium can release ATP in response to different stimuli [87–91]. The absence of retinal pigment epithelium in E6.5 retina explants rules this out as a possible source of ATP, but Müller cells could produce this nucleotide in our experimental system. Müller glial cells are the main astrocytic cell type in the retina of birds, which are devoid of astrocytes. Each Müller cell constitutes the core of a column of retinal neurons, which represents the smallest functional unit of the retina [92]. Müller cells interact with the neurons in their columns in a type of symbiotic relationship, and they are responsible for the functional and metabolic support of their associated neurons. Among their functions, Müller cells provide neurons with trophic substances and remove metabolic waste, they are responsible for the homeostatic and metabolic support of retinal neurons, and they also play a critical role in the regulation of: extracellular space volume; ion and water homeostasis; synaptic activity, by neurotransmitter recycling; and the release of gliotransmitters [92]. In this context, it has been observed that Müller cells from E8 chick embryo retina (developmental age equivalent to E7 in the quail) release ATP *in vitro* [93]. Given that microglial cells a migrate tangentially in the embryonic quail retina on Müller cell endfeet [15], the production of ATP by Müller cells might induce the chemokinesis of microglial cells. In a previous study, we found that Müller cells can phagocytose apoptotic cells in the embryonic quail retina [16]. Müller cells are activated by numerous stimuli, including cell death, and might therefore be activated by developmental cell death, possibly releasing ATP that might induce the chemokinesis of microglial cells. However, further research is required to test this tentative hypothesis.

Finally, it appears evident that other chemotactic molecules must contribute to regulating the entry and migration of microglial precursors into the retina. In support of this possibility, lysophosphatidylcholine and the chemokine CXCL12 were recently found to mediate recruitment of microglial cells into the developing zebrafish brain and the subventricular zone of the developing mouse cerebral cortex, respectively [31, 45].

In the present study, only relatively high concentrations of either ATP (1 mM) or UDP (1 mM) had a significant effect on the entry of microglial precursors into the retina, probably because of the rapid hydrolysis of these nucleotides into nucleosides, including adenosine, by ectonucleotidases [57, 58, 94], some of which are known to be expressed by microglial cells [95]. Therefore, microglial cells could be exposed to both nucleotides and nucleosides [58] and sense both signals, because they possess multiple P1 (selective for adenosine) and P2 (sensitive to nucleotides) purinergic receptors [41, 43]. Indeed, co-stimulation of both P2Y and P1 receptor subtypes was found to be required for chemotaxis of purified microglia from newborn mouse brain towards an ATP source [58]. Retina explants were treated with the non-hydrolyzable ATP analogue ATPγS to test whether ectonucleotidases acted in our *in vitro* system. Treatment with 100 μM ATPγS induced a similar increase in the entry and elongation of microglial cells to that observed after explant treatment with 1 mM ATP. These findings suggest that ATP has a direct effect on the entry of microglial cells and that a certain amount may be hydrolyzed to AMP or adenosine.

In conclusion, this study offers experimental evidence of a relationship between retinal cell death and the entry of microglial cells into the developing quail retina. In addition, the findings indicate a major role for purinergic signaling in the early entry and subsequent migration of microglial precursors in the retina. Our results demonstrate that extracellular ATP and UDP exert a chemokinetic effect on microglial cells entering the retina through purinergic receptors, probably of P2Y subtype. Nucleotide signaling also participates in the recruitment of microglial progenitors into the developing zebrafish brain [44], suggesting a generalized mechanism of microglial recruitment in the developing CNS of vertebrates.
